# Resolving the apparent paradox of multisensory expertise: coupling priors, common-cause priors, and how to distinguish among them

**DOI:** 10.3389/fnhum.2026.1960361

**Published:** 2026-10-29

**Authors:** Alessandro Bortolotti, Riccardo Palumbo, Fabio Lokwani-Di Matteo, Pier Luigi Sacco

**Affiliations:** 1 Department of Neuroscience, Imaging and Clinical Sciences, University “G. d'Annunzio” of Chieti-Pescara, Chieti, Italy; 2 metaLAB (at) Harvard, Cambridge, MA, United States

**Keywords:** causal inference, common-cause prior, coupling prior, cross-modal correspondences, multisensory integration, parameter recovery, perceptual expertise, temporal binding window

## Abstract

Perceptual expertise is widely assumed to refine multisensory perception, yet the empirical literature appears inconsistent. Trained musicians show narrower audiovisual temporal integration windows and quicker segregation, whereas experienced wine tasters seem more influenced by a wine's color in their aroma and flavor judgments than novices are. We argue that the inconsistency arises from a conflation of two formally distinct quantities: the prior probability that concurrent signals arise from a common source, and the coupling between the values of signals that share one. We present a generative model in which both are free parameters, along with channel precision and a decision criterion, that contains causal-inference and coupling-prior models as special cases. The model shows that the correlation between the values of two signals across an environment reflects only the product of the two priors, so that a correlation-tracking learner acquires the coupling when the signals always share a source, whereas separating the two priors requires representing causal structure; that binary common-source reports confound the common-cause prior with the decision criterion; that the coupling trades off against the measurement noise of the influencing channel unless its precision is measured independently; and that the two priors leave distinct signatures in how cross-modal influence varies with disparity. When the source is fixed, as for the color and odor of a wine, tighter coupling alone produces the conjunction of greater displacement by color and better discrimination between wines reported for experienced tasters, which no change in odor precision can produce. Simulations of a test battery combining continuous estimates, common-source reports and discrimination thresholds show that the parameters can be recovered, that a within-subject dissociation can be detected with conventional sample sizes under illustrative effect sizes, and that recovery fails in certain cases. The account yields a rule classifying environments by their base rate of common causation and their coupling strength, and the same ambiguity attends claims about altered multisensory processing in autism and aging. We also show that the most cited demonstration of expert susceptibility to color contains no expertise manipulation, and suggest that apparent expert biases may reflectwind calibration to real-world statistics.

## 1 Introduction

Musicians detect audiovisual asynchrony at a finer temporal grain than non-musicians, at least for musical material, and the width of their temporal integration window scales with the amount of recent practice (Lee and Noppeney, 2011, 2014). Short-term perceptual training produces the same narrowing in naive observers (Powers et al., 2009). The natural summary of this work is that expertise buys perceptual independence, so that the expert holds the channels apart where the novice lets them merge.

Set beside it is a second and much thinner body of work in which expertise appears to do the opposite. Wang and Spence (2019) asked 168 participants, stratified by wine experience, to describe a white wine, a rosé, and the same white wine dyed to match the rosé. Only the participants with tasting experience were displaced: intermediates and experts judged the dyed white to be far more similar in aroma to the genuine rosé than to the chemically identical undyed white, whereas beginners showed no such displacement. Summarizing the literature, Spence (2020) proposes that the subtle gradations of color in wine carry more meaning, and therefore set more specific flavor expectations, for experts than for beginners. Here the expert is the one who lets the channels merge.

Box 1 **Terms used in the text.**

**Common-cause prior (p**_**C**_**)**. The prior probability that two concurrent signals arise from a single source; in causal-inference models, the mixture weight over causal structures (Körding et al., 2007).

**Coupling prior (ρ, w)**. A joint prior over the values of two signals, concentrated along their learned relation (Ernst, (2005, 2007). In earlier models, it applies to every pair of signals and its width also governs how far they are combined; here it describes the values of signals that arise from one source and whether they belong to p_C_. ρ is the correlation between the values and w is the standard deviation of their difference, so that the coupling is tighter as ρ rises and w narrows.

**Channel precision (π_i_)**. The inverse variance of the noise with which a channel measures its attribute, independent across channels. Precision concerns the noise around each measurement, whereas the coupling concerns the covariance of the attribute values themselves; both belong to the generative model.

**Decision criterion (κ)**. The posterior probability of a common source above which an observer reports one.

**Unity assumption**. The belief that two signals refer to one event or object; Chen and Spence (2017) propose quantifying this as the prior probability of a common source; the belief formed on a given trial corresponds to its posterior.

**Cross-modal correspondences**. Consistent mappings between attribute values in different senses that, in these terms, act on the coupling between values and, when they signal that two stimuli belong together, on the belief in a common source.

**Semantic congruency**. Agreement in identity or meaning between the signals, such as a bark and the image of a dog, which supports the belief that they share a source.

**Simultaneity judgment, modeled and unmodeled**. A report of whether two signals were simultaneous; unmodeled when the proportion of synchrony responses is summarized directly, which confounds sensory noise, the prior, and the criterion, and modeled when an observer model separates them (O'Donohue et al., 2022).

**Illusion susceptibility**. The rate at which a conflict between signals produces an illusory percept, such as the double flash or the McGurk fusion, which depends jointly on the priors, the precisions and the criterion; a temporal illusion is one in which a signal in one sense alters the perceived timing or number of events in another.

**Selection**. The possibility that experts differ from novices because people with particular perceptual characteristics are drawn to a domain.

Both patterns are conventionally glossed as expertise refining perception, but the gloss cannot cover both. Chen and Spence (2017) come close to the point, noting that the unity assumption, cross-modal correspondences, and semantic congruency are difficult to separate empirically (Box 1 defines these and the other terms on which our argument relies). The specific conflation of temporal binding precision with cross-modal influence between attribute values has not, to our knowledge, been named as such. The observation has not been taken up, and the tension it identifies remains, to our knowledge, unresolved.

We propose that the tension is an artifact of a conflation embedded in the field's terminology. Two quantities are routinely treated as one parameter under two names. The first is the prior probability that concurrent signals arise from a single source, the scalar mixture weight of causal-inference models (Körding et al., 2007; Shams and Beierholm, 2010). The second is the coupling prior in the sense of Ernst (2007), a joint prior density over signal values whose correlation determines how strongly one channel's estimate pulls on the other's. These encode different facts about an environment because they capture different features of the same joint signal statistics. We will argue that they require different statistical information to learn and are plausibly implemented by different neural mechanisms. Each quantity has a place in a model the field already uses, and both models have been applied to the same phenomena of partial integration: causal-inference models represent the probability of a common source and treat its values as identical, whereas coupling-prior models represent how tightly the values are statistically related and leave causal structure unrepresented, the width of the prior carrying the whole question of how far two signals are combined. What we add is a single generative model in which both are free, an analysis of the conditions under which each can be identified from behavior, and a rule that predicts which of them is adjusted by expertise. Once they are held apart, the musician and the taster are seen to have learned different things, and the apparent contradiction dissolves without either result having to be discounted.

The separation also provides what a purely descriptive taxonomy could not: a rule for classifying an environment that the framework has not already seen. A trained environment is characterized by two statistics that vary independently: the base rate with which concurrent signals in that environment share a source, and the strength of the correlation between their values when they do (Figure 1A). The ensemble musician inhabits an environment of high correlation and low base rate, since a great many sounds and movements co-occur in an orchestra and few pairs of them belong to one act. The taster inhabits an environment where the base rate is fixed at unity, since there is one glass and nobody maintains the hypothesis that the color and the odor come from different objects. Hence, the correlation is the only quantity left to learn.

**Figure 1:**
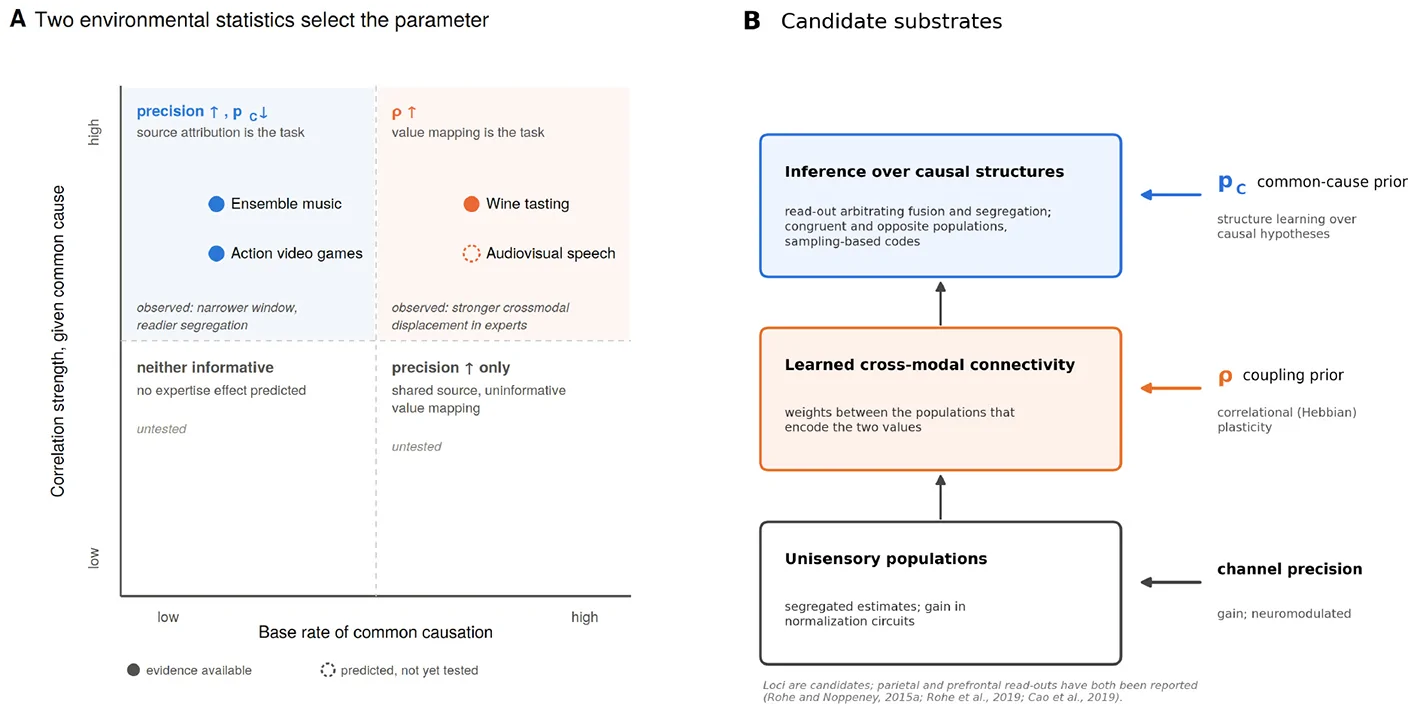
Two priors, two environmental statistics, and their candidate substrates. **(A)** Environments vary independently in the base rate with which concurrent signals share a source and in the strength of the correlation between their values given a shared source. The two statistics select which parameter expertise adjusts. Ensemble music couples a high correlation to a low base rate, so precision rises and the common-cause prior falls; wine tasting fixes the base rate at unity, leaving the coupling prior as the only free quantity. The lower quadrants generate predictions for cases not yet studied. **(B)** Candidate substrates: channel precision as gain in normalization circuits of unisensory populations; the coupling as learned cross-modal connectivity between the populations that encode the two values; the common-cause prior in the read-out that arbitrates betwe en causal structures, for which congruent and opposite populations and sampling-based codes have been proposed. The loci are candidates, and the model constrains the computation more tightly than it constrains the anatomy.

We have ourselves contributed to the descriptive tradition and to its limitations. In earlier work we proposed that certain culturally elaborated experiences engage multiple processing streams in ways that resist decomposition, and that expert practitioners process modalities in parallel with continuous cross-modal interaction, whereas novices process sequentially before integrating (Bortolotti et al., 2025). That proposal was stated without committing to an integration model or deriving a testable prediction, and the present study attempts to do both.

The conflation we diagnose is not peculiar to musicians and tasters, and the clearest sign that it matters comes from a literature that has already run into it without naming it explicitly. Work on multisensory perception in autism spectrum disorder has established that cue combination itself is unimpaired: Zaidel et al. (2015) found visual-vestibular integration in autistic adolescents to match optimal predictions even under noise, whereas the same participants showed heightened sensitivity to sensory noise and an inflexible, non-experience-dependent use of prior knowledge. Noel et al. (2022) subsequently fitted a causal-inference model to audiovisual data from autistic participants. They reached a similar conclusion, finding integration compatible with optimal cue combination together with differences in the prior probability of a common cause and in choice biases. Both results dissociate precisely the kind we propose, in which cue combination is intact and a parameter of the prior differs, and neither has been described as such. It must be remarked that the broader Bayesian account of autism remains contested (Angeletos and Seriès, 2023), which is itself an argument for specifying which parameter is at issue. A comparable ambiguity concerns healthy aging, where systematic review evidence points specifically to a failure to weight sensory information appropriately rather than to a failure to integrate (de Dieuleveult et al., 2017), and where the parameter carrying the change has not been identified. The same question recurs wherever causal inference is invoked to explain how the multisensory perception of one population differs from that of another: which of two formally distinct parameters an observed difference belongs to.

The remainder of the paper is organized as follows. Section 2 reviews the two bodies of evidence and shows that the most frequently cited demonstration in the second contains no expertise manipulation. Section 3 states a generative model in which the two priors, the precision of each channel and a decision criterion, are separate parameters, derives what each contributes to responses, and shows that a tighter coupling suffices to produce the chemosensory evidence. Section 4 develops the classification rule and the learning argument, and confronts the results that appear to threaten the account together with the single-parameter rival. Section 5 reconsiders the chemosensory case, distinguishing calibration from bias and a perceptual from a decisional locus of the coupling. Section 6 discusses candidate implementations, Section 7 reports simulations that test whether the parameters can be recovered from a realistic test battery, Section 8 sets out predictions and an empirical program, and section 9 discusses limitations and concludes.

## 2 Two expertise effects that appear to point in opposite directions

### 2.1 Sharpening: temporal precision and readier segregation

The best-documented effect of expertise on multisensory processing concerns the temporal window within which signals from different modalities are treated as belonging to a single event. Lee and Noppeney (2011) had amateur pianists and non-musicians judge the synchrony of audiovisual piano performance and audiovisual speech and found that the musicians' integration window was significantly narrower for music. Their follow-up added sine wave analogs of speech and found the narrowing largest for music, present for sine wave speech, and graded across stimulus classes, with the window for music decreasing with the amount of recent practice (Lee and Noppeney, 2014). Comparable narrowing has been reported with simple non-musical stimuli (Bidelman, 2016), and musicians show enhanced audiovisual temporal discrimination at fixed asynchronies (Lu et al., 2014). Short-term perceptual training in naive observers produces the same effect (Powers et al., 2009), and the one controlled training study found narrowing only in the trained group (Che et al., 2022). Musicians have also been reported to resist the McGurk effect (Proverbio et al., 2016).

It must be remarked, however, that this literature is considerably less secure than its citation frequency suggests, and four qualifications bear directly on the argument that follows.

First, the domain specificity of the founding result is routinely lost in citation. Lee and Noppeney (2011) found narrowing for music and not for speech, and their own interpretation appeals to a motor forward model specific to piano playing rather than to any general recalibration of multisensory binding. The 2014 follow-up reports partial generalization, though the extension to natural speech rests on a one-tailed test at *p* = 0.07.

Second, a substantial part of what is measured as window width may be a decision criterion rather than perceptual binding. O'Donohue et al. (2022) fitted an observer model to simultaneity judgments that separates decisional boundaries from sensory noise. They found musicians differed on both, with the reduction in sensory noise carrying the larger effect. Since almost every musician result in this area derives from unmodeled simultaneity judgment or from illusion susceptibility, the field's central parameter is less cleanly identified than it appears, and we impose the observer-model requirement on our own proposed designs in Sections 7 and 8 accordingly.

Third, stimulus acoustics may account for a share of the apparent expertise effects. Bishop and Goebl (2014) tested pianists, clarinetists, and violinists; they found that all groups performed best on piano and worst on violin regardless of their own training, with some evidence of an instrument-specific advantage only for pianists. Piano onsets carry sharp attack transients and violin onsets do not, and Lee and Noppeney used piano stimuli.

Fourth, and most consequentially for what follows, expertise improves single-channel sensitivity as well. Rammsayer et al. (2012), testing 40 musicians and 40 non-musicians, found musicians superior on four of six timing tasks spanning both the auditory and the visual modality, with no group difference on either temporal-generalization task. Korczyk et al. (2022) report a 16 percentage-point advantage for pianists on a purely visual rhythm discrimination task. Our account accommodates this, since raised channel precision is one of the parameters we consider. It creates a difficulty of a different kind, taken up in Section 4.4: raising precision alone, with the prior of common cause held fixed, narrows the integration window, produces readier segregation and improves unimodal timing, and it reduces cross-modal capture at small disparities whenever the coupling between the two values is loose relative to sensory noise (Section 3.4). Much of the musician profile can therefore be produced by precision alone, and Section 4.4 asks whether a second parameter is needed on the musician side.

It is also important to point out discordant results here. Jicol et al. (2018) report that musicians and drummers recalibrate simultaneity judgments in the direction opposite to non-musicians following adaptation, whereas O'Donohue et al. (2022), in a better-modeled design, found no group difference in recalibration. More consequentially, the McGurk result is not corroborated by the one study to have examined it with graded measures of training: Lee et al. (2024) did not detect an association between McGurk susceptibility and graded musical experience in an unselected sample of 73 adults. We return to that null in Section 4.1, where the framework predicts it.

Finally, the most informative single result on the shape of the expert profile comes from Landry et al. (2017), who tested musicians on audiotactile tasks and found an advantage confined to the temporal illusion, with no detected group difference on task-relevant unisensory abilities or on the non-temporal parchment-skin illusion. What transfers, on their reading, attaches to a type of inferential problem rather than to a pair of modalities.

A recent causal-inference fit to musicians bears directly on the parameters at issue. In a preregistered comparison of 35 musicians and 35 non-musicians localizing flashes and noise bursts, musicians showed reduced ventriloquism, captured by a lower prior of common cause, with no detected group difference in fitted sensory precision (O'Donohue et al., 2026). The effect appears in a spatial task outside the musicians' training, consistent with the reading that what transfers is tied to a type of inferential problem (Landry et al., 2017), here the attribution of concurrent signals to sources, and compatible with the failure of individual differences in common cause priors to generalize across tasks (Odegaard and Shams, 2016) if those differences are partly task-specific.

### 2.2 Amplification: cross-modal influence in the chemical senses

The strongest available demonstration on the other side, which we treat as an illustrative case for the reasons given below, is that of Wang and Spence (2019), whose design is described above. The descriptor-profile similarity between the genuine rosé and the dyed white reached 0.89 for both intermediates and experts on smell, compared with 0.59 for beginners; on smell alone, the two experienced groups rated the rosé and the dyed white as more similar to each other than the rosé and the undyed white. A participant-level reanalysis of the public data, which reproduces these coefficients (Supplementary material S4), gives an expert-minus-beginner difference of 0.32 in how much closer the dyed white's aroma profile lay to the rosé than to the white, with a 95% bootstrap interval from −0.04 to 0.64 (Efron, 1979), and of 0.40 (0.10 to 0.72) with the seven published descriptor categories. Parr et al. (2003) hypothesized the same direction. They reported that wine experts discriminated the masked wines while still succumbing to some color-induced olfactory bias, whereas social drinkers in a separate experiment responded indiscriminately in some conditions.

This evidence, however, requires qualification. The most frequently cited study in this area cannot support any claim about expertise. Morrot et al. (2001) are often cited as showing that wine experts, when presented with a white wine dyed red, described it in the vocabulary of red wine. The tasting experiment included 54 undergraduates from a faculty of enology; the study mentions nothing about experts, novices, or untrained participants. It includes no novice comparison group, no measure of expertise, and no expertise factor in the design. The four professionals in the study are authors of published tasting notes that underwent lexical analysis, and no experiment was conducted with them. No inferential statistics are reported for the tasting results. We cite the study only as the source of the field's framing, for reasons that Section 5.1 makes explicit.

The two studies that do compare experienced with inexperienced tasters share a confound. In both, the inexperienced group was largely undifferentiated across all stimuli, including the ones that did differ: beginners in Wang and Spence rated the smell profiles of the rosé and the white as 0.64 similar, against 0.43 and 0.48 for the experienced groups. A smaller measured color effect in a group that discriminates poorly across the board is confounded with a discriminability floor, and neither study tested the expertise by color interaction directly. In the former, expertise was self-reported, and the contrast on flavor, where beginners reached 0.83 against 0.88 and 0.95, is considerably weaker than the contrast on smell. Nor is the direction uncontested: Ballester et al. (2009) found that experts and novices alike categorized red and white wines, though not rosés, by odor alone, with no difference between the groups, and Valentin et al. (2016) found color not to be a major factor in professional assessment of Pinot noir. A reader who judges this evidence too weak to bear a theoretical framework is not being unreasonable, and we note in Section 9 what survives if the amplification effect proves null.

However, one pattern in these data a change in the reliability of the chemosensory channel cannot produce. It has gone unremarked: the experienced groups in Wang and Spence discriminated the wines that differed in composition better than beginners while being displaced by color more, and both judgments were made with the color of each wine visible. A gain in odor precision improves discrimination and reduces displacement, whereas a tighter learned mapping between hue and odor improves both at once, because the hue of a rosé is itself informative about its odor, a result derived formally in Section 3.5. This is the observation that motivates what follows.

### 2.3 A diagnosis

The two literatures measure different quantities in different paradigms and report them under a shared description. Table 1 lists the studies on which each rests, along with their participants, paradigms, and measures. Simultaneity judgment, temporal order judgment, and the double-flash illusion assess the resolution and criteria by which the system decides whether concurrent signals belong to one event, a question about source attribution. Descriptor generation and identification under cross-modal conflict assess how strongly the value of one channel constrains the interpretation of another once the signals are taken to belong together, a question about the learned mapping between values. Treating both as expertise sharpening multisensory perception has obscured the fact that they move in opposite directions, and the field has no account of why they should. We propose that they concern two parameters.

**Table 1:** Studies on which the two expertise effects rest.

Study	Participants	Paradigm and measure	Main result	Quantity implicated
Lee and Noppeney (2011)	Amateur pianists and non-musicians	Synchrony judgments, audiovisual piano music and speech	Narrower integration window for music, not for speech	Window width (unmodeled)
Lee and Noppeney (2014)	Musicians and non-musicians	Synchrony judgments, music, sine wave speech and speech	Narrowing graded across stimulus classes; window for music decreasing with recent practice	Window width (unmodeled)
Bidelman (2016); Lu et al. (2014)	Musicians and non-musicians	Double-flash illusion; audiovisual temporal discrimination	Narrower binding with simple stimuli; better discrimination at fixed asynchronies	Window width; precision
Powers et al. (2009); Che et al. (2022)	Naive observers; randomized music lessons	Perceptual training; audiovisual temporal processing	Narrowing after training; narrowing only in the trained group	Window width
O'Donohue et al. (2022)	Musicians and non-musicians	Simultaneity judgments fitted with an observer model	Lower sensory noise, the larger effect, and different decisional boundaries	Precision and criterion
Bishop and Goebl (2014)	Pianists, clarinetists, violinists	Audiovisual synchrony across instruments	All groups best on piano; some evidence of an instrument-specific advantage only for pianists	Stimulus acoustics
Rammsayer et al. (2012); Korczyk et al. (2022)	Musicians and non-musicians; pianists	Unimodal auditory and visual timing; visual rhythm discrimination	Musicians better on four of six tasks; pianists better on visual rhythm	Unimodal precision
Proverbio et al. (2016); Lee et al. (2024)	Musicians; large unselected sample	McGurk illusion	Reduced illusion in musicians; no relation to musical experience	Illusion susceptibility
O'Donohue et al. (2026)	35 musicians and 35 non-musicians (preregistered)	Audiovisual spatial localization fitted with a causal-inference model	Less ventriloquism in musicians, captured by a lower common-cause prior with precision matched; instruction lowered the prior without removing the group difference	Common-cause prior
Landry et al. (2017)	Musicians and non-musicians	Audiotactile illusions and unisensory tasks	Advantage confined to the temporal illusion	Type of inferential problem
Wang and Spence (2019)	168 participants stratified by wine experience	Descriptors of a white, a rosé, and a dyed white, color visible	Experienced groups displaced by color, with greater separation of the descriptor profiles of rosé and white	Coupling of hue and odor
Parr et al. (2003)	Wine experts; social drinkers in a separate experiment	Aroma judgments of masked and colored wines	Experts discriminate yet show some color-induced bias	Coupling (mixed evidence)
Morrot et al. (2001)	54 undergraduates in enology, no expertise contrast	Descriptor allocation between a white and the same wine dyed red	Allocation follows color	None (no expertise factor)
Ballester et al. (2009); Valentin et al. (2016)	Experts and novices; professionals	Odor-based categorization by color class; assessment of Pinot noir	Red and white, not rosé, wines categorized by odor, with no expertise effect; color not a major factor	None detected
Croijmans and Majid (2016); Croijmans et al. (2020)	Wine and coffee experts; wine trainees	Smell and taste identification; imagery vividness	No identification advantage; more vivid imagery after training	Semantic or perceptual (undistinguished)

Window width refers to integration windows estimated from the proportion of synchrony responses without an observer model, which confounds sensory noise, the prior, and the decision criterion (Box 1).

## 3 A generative model with two priors

### 3.1 The model

The vocabulary of the field presents two quantities as alternatives for a single construct. Chen and Spence (2017) write of “coupling priors” or the “prior of common cause” as ways of quantifying the unity assumption as a continuous variable, and the usage is standard. The two are formally related, since a mixture coupling prior with a broad tail behaves much like a two-component causal-inference model (Roach et al., 2006), and models of the two kinds have been shown to account for overlapping data (Sato et al., 2007). They are non-etheless distinct objects, as the model stated below makes explicit.

The prior of common cause, which we write p_C_, is a scalar mixture weight over causal structures in the model of Körding et al. (2007). It answers the question of whether the two signals arise from one source or from two, and it produces the characteristic non-linear breakdown of fusion at large disparity: below some disparity the estimates fuse, above it they segregate, and p_C_ sets where the transition falls. It encodes a base rate, namely how often, in this environment, concurrent signals of these kinds turn out to share a source.

The coupling prior of Ernst (2005, 2007) is a joint prior density over the values of two signals, concentrated along their learned relation, whose width determines how strongly each channel's estimate pulls on the other's. Ernst induced one experimentally by training an arbitrary but consistent mapping between a visual and a haptic signal, and observers who had learned the mapping subsequently integrated the two in a way they had not before. In that study, the coupling prior applies to every pair of signals and carries two roles at once: it encodes how the values covary, and its width also governs how far the signals are combined, which is how it accounts for partial integration without representing causal structure (Ernst, 2005; Bresciani et al., 2006). The present model separates the roles. The coupling, a proper bivariate Gaussian with correlation ρ, describes how the values of causally related signals covary, a regularity in the world, and whether the two signals are causally related is assigned to p_C_, so that each role can be learned from its own statistics (Section 3.3) and identified from behavior (Section 3.4).

We consider an observer who receives two signals, each of which carries the value of an attribute: the time of an auditory and of a visual onset, the location of a sound and of a flash, or the hue and the odor of a wine, expressed on a common scale once the learned mapping between them has been applied. We write s_1_ and s_2_ for the attribute values and x_1_ and x_2_ for their noisy measurements. A binary causal structure C takes the value 1 when the two signals arise from a single source and 2 when they arise from independent sources, and the prior probability of a common source is p_C_ = P (C = 1). Given a common source, the two values are jointly Gaussian with marginal variance $\sigma_{\text{p}}^{2}$ and correlation ρ, so that the coupling width w, defined as the standard deviation of the difference s_2_ – s_1_ under a common source, satisfies w^2^ = 2$\sigma_{\text{p}}^{2}$(1 – ρ). Given independent sources, the two values are independent and have the same marginal variance, so the causal structure leaves the distribution of each value unchanged and alters only their relation. Each measurement adds Gaussian noise of variance $\sigma_{\text{i}}^{2}$, whose inverse is the precision π_i_ of channel i. The generative model can be written compactly as


$$\begin{array}{ccc} P\left(C=1\right) & = & p_{C},\;\left(s_{1},s_{2}\right)|C=1\sim N\left(0,\Sigma_{1}\right)\\ \left(s_{1},s_{2}\right)|C & = & 2\sim N\left(0,\Sigma_{2}\right)\\ \Sigma_{1} & = & \sigma_{p}^{2}\left(\begin{array}{cc} 1 & \rho \\ \rho & 1 \end{array}\right),\;\Sigma_{2}=\sigma_{p}^{2}I,\\ x_{i} & = & s_{i}+\varepsilon_{i},\;\varepsilon_{i}\sim N\left(0,\sigma_{i}^{2}\right) \end{array}$$ (1)

where R = diag ($\sigma_{1}^{2}$, $\sigma_{2}^{2}$) collects the measurement noise, which is independent across the two channels. The coupling ρ is therefore a correlation between the attribute values themselves, a covariance of the signals in the world, and the measurements of the two channels are correlated only through it. The observer inverts this model, and the log posterior odds of a common source are


$$L\left(x\right)=\text{logit}p_{C}+\log N\left(x;0,\Sigma_{1}+R\right)-\log N\left(x;0,\Sigma_{2}+R\right)$$ (2)

where “logit” is the log odds of a probability: logit *p* = log[p/(1 – p)], while the estimates of the two values average the estimates obtained under each structure, weighted by the posterior probability of that structure:


$$\begin{array}{lll} \hat{s} & = & P(C=1|x)\Sigma_{1}{\left(\Sigma_{1}+R\right)}^{-1}x \end{array}\begin{array}{l} +[1-P(C=1|x)]\Sigma_{2}{\left(\Sigma_{2}+R\right)}^{-1}x \end{array}$$ (3)

This read-out, known as model averaging, describes audiovisual numerosity judgments better than model selection and probability matching at the group level (Rohe et al., 2019), whereas probability matching describes many individual observers in spatial localization (Wozny et al., 2010). A categorical report that the two signals share a source is issued when P(C = 1 | x) exceeds a criterion κ. The questions of whether expertise changes a prior, the precision of a channel, or a criterion of report thus receive separate answers within one model, which contains two priors (p_C_ over causal structures and ρ over attribute values given a common source), a likelihood (the precisions π_i_) and a decision rule (κ).

### 3.2 What the established models fix

The model contains the principal accounts of partial integration as special cases (Proposition 1): with ρ = 1 it is the causal-inference observer, with p_C_ = 1 an observer with a Gaussian coupling prior, and with both it is forced fusion. The five formal results are stated and proved in Supplementary material S1 and cited here by number with their content. These accounts were proposed as alternative solutions to the same correspondence problem, in place of forced fusion (Ernst, 2005; Körding et al., 2007; Shams and Beierholm, 2010), and they have been compared directly as accounts of human performance (Körding et al., 2007; Rohe and Noppeney, 2015b). When ρ = 1, the coupling width is zero, the values coincide under a common source, and Equations 2, 3 reduce exactly to the causal-inference observer of Körding et al. (2007), with identical likelihoods and estimates. When p_C_ = 1, the observer holds a single bivariate Gaussian prior over the two values, and the estimates are drawn toward each other by an amount that w sets. This is the Gaussian coupling prior of Ernst (2005, 2007) and Bresciani et al. (2006), exactly in the limit σ_p_ → ∞ with w fixed, where the prior becomes flat along the identity line as in their formulation, and otherwise up to a common shrinkage of both estimates toward the prior mean (Supplementary material S1). When p_C_ = 1 and ρ = 1, the model reduces to forced fusion. The prior proposed by Roach et al. (2006), a Gaussian ridge along the diagonal added to a constant, has the same structure of integration and segregation, which the family approximates when its independent component is broad, and integrating the causal structure out of the causal-inference model yields exactly the member of the family with ρ = 1, a ridge of zero width combined with an independent component (Körding et al., 2007; Shams and Beierholm, 2010).

The two parameters therefore span the space of models the field has used, and distinguish between them. Read through the present family, the models fitted in practice occupy its two limits. Causal-inference models set the coupling at unity, which suits the redundant attributes on which those models were developed, since the location or the time of a single event is one quantity that two senses measure. A common source then entails identical values. Coupling-prior models make no statement about causal structure, and in the present family they occupy the limit p_C_ = 1, where the coupling width accounts for all the partial integration that the data show. In either case, a difference between groups is attributed to the one free parameter that the fitted model contains. The causal-inference model owes its advantage in fitting behavior to adapting its effective coupling to each stimulus via the posterior over causal structures, whereas a fixed coupling prior achieves this only with a different prior for each environment (Ernst, 2007). Our model inherits this adaptivity. For non-redundant attributes, such as the hue and the odor of a wine, a common source does not entail identical values, and the concentration of the values around their learned relation becomes a quantity in its own right. Our proposed hypothesis is that expertise can move the two parameters separately, according to the statistics of the environment in which it is acquired.

That the two can move independently is not merely a formal possibility. Beierholm et al. (2009) found that the estimated priors remained stable when the likelihoods were changed with two very different sets of visual stimuli, which suggests that priors are encoded independently of likelihoods. It must be remarked that this is not quite the independence that our account requires, since the two quantities we separate are both priors. Their mutual independence has not to our knowledge been tested directly, a test whose feasibility is examined in Section 7. Ernst and Bülthoff (2004) draw the related distinction between sensory *combination*, in which non-redundant signals in different units are merged, and sensory *integration* proper, in which redundant estimates in a common metric are averaged with reliability weights. The distinction matters here because the wine case belongs to the first category and the audiovisual timing case to the second.

### 3.3 What can be learned from an environment

The two parameters are properties of the same joint distribution of attribute values, and they are different properties of it: p_C_ is the weight of the component in which the values are concentrated around their relation, and ρ is the degree of that concentration. The first consequence is that the familiar second-order statistic of an environment carries only their product (Proposition 2). The correlation between the two values across events in an environment equals p_C_ ρ, and so does the slope of the regression of either value on the other. As a result, an environment with frequent common sources and loosely related values can yield the same correlation as an environment with rare common sources and tightly related values. A learner that tracks second-order statistics, such as a covariance or delta-rule learner operating on the two values, converges to the product. In two simulated environments matched on the product, one with rare common sources and a tight coupling (*p*_*C*_ = 0.3, ρ = 0.95) and one with a common source on every event (*p*_C_ = 1, ρ = 0.285), such a learner converged to 0.28 and 0.29 (Supplementary Figure S2). These statistics concern the values themselves, which the simulated learners observed directly. Noisy measurements have the same covariance, since the noise is independent across channels, but variances larger by $\sigma_{1}^{2}$ and $\sigma_{2}^{2}$, so that their correlation is attenuated and the kurtosis of their difference diluted; the two priors remain recoverable once the noise variances are known, as the unimodal measures of Section 7 provide (Supplementary material S1). The two factors are separated by the shape of the distribution. The difference s_2_ – s_1_ is a scale mixture of two Gaussians, whose excess kurtosis


$$\begin{array}{l} \gamma_{2}=\frac{3p_{C}(1-p_{C})\rho^{2}}{{\left(1-p_{C}\rho \right)}^{2}} \end{array}$$ (4)

vanishes when p_C_ = 1 and, once the product c = p_C_ ρ is known and lies strictly between 0 and 1, identifies the base rate through p_C_ = 3c^2^/[3c^2^ + γ_2_(1 – c)^2^]. A maximum-likelihood learner recovered *p*_C_ = 0.30 and ρ = 0.95 in the first environment and *p*_C_ = 1.00 and ρ = 0.30 in the second, where the base rate was estimated less precisely (interquartile range from 0.92 to 1.00), since a loose coupling makes the two causal structures similar.

When the base rate is fixed at unity, as for the wine in the glass, the product equals ρ and correlational learning suffices to acquire the coupling; when it is below unity, as for the gestures and sounds of an ensemble, correlational learning confounds a low base rate with a loose coupling, and separating the two requires a learner that represents the mixture of causal structures (Section 4.2).

### 3.4 How the components shape responses

Three identification results, stated in Propositions 3 and 4, organize the design of the test battery examined in Section 7. The first concerns the criterion: since p_C_ enters the log posterior odds (2) only as an additive constant, any report that compares the posterior with a criterion depends on p_C_ and κ only through the difference logit p_C_ – logit κ, and this holds whatever the likelihoods. Binary judgments of simultaneity or of common source therefore cannot separate the common-cause prior from the criterion of report, whereas continuous estimates, in which the posterior enters as a weight in (3), identify p_C_ in designs such as that of Section 7. The second concerns the coupling and the precision of the influencing channel. When the prior over values is broad relative to the noise, the weight of x_2_ in the estimate of s_1_ under a common source tends to $\sigma_{1}^{2}$/($\sigma_{1}^{2}$ + $\sigma_{2}^{2}$ + w^2^), and the log posterior odds tend to


$$\begin{array}{l} \begin{array}{lll} L & \to & \text{logit}p_{C}+\frac{1}{2}\log\frac{\sigma_{p}^{2}}{V_{1}}-\frac{{\left(x_{2}-x_{1}\right)}^{2}}{2V_{1}}, \end{array}\\ \begin{array}{lll} V_{1} & = & w^{2}+\sigma_{1}^{2}+\sigma_{2}^{2} \end{array} \end{array}$$ (5)

The coupling width and the noise of the influencing channel thus enter the estimate of the other channel only through their sum, and they can be separated only by measuring each channel's precision on its own, preferably with a method that does not mix sensory noise with the noise of report, such as a discrimination threshold. The same expression shows that p_C_ shares the intercept of the log-odds with the width of the prior over values, which the central tendency of unimodal reports must pin down. The third result concerns the two priors themselves, which act on different features of the dependence on disparity: p_C_ shifts the log-odds uniformly, whereas the coupling changes both their curvature and the weight applied under a common source, and the two separate when a design samples disparities across the region in which integration gives way to segregation.

These properties produce distinct signatures in how one signal influences the estimate of the other (Figure 2). In the baseline regime of Figure 2, lowering p_C_ from 0.5 to 0.3 reduced the influence at small disparity from 0.33 to 0.21 and shortened its range, whereas halving the coupling width raised the influence to 0.39 and shortened its range. Across 150 random draws of the parameters, a change in p_C_ moved the amplitude and the range of the influence in the same direction in every draw, a change in the coupling moved them in opposite directions in every draw, and a change in the precision of the influencing channel alone acted as a change in the coupling in every draw. A joint increase in the precision of both channels narrowed the range in every draw, whereas its effect on the amplitude depended on the coupling: it lowered the amplitude, as a lower p_C_ does, whenever the coupling width exceeded about half of the combined sensory noise ($\sigma_{1}^{2}$ + $\sigma_{2}^{2}$)^1/2^, and it raised the amplitude, as a tighter coupling does, whenever the coupling width fell below about a quarter of that noise. Precision is thus confounded with one prior or with the other in every regime, unless it is measured independently. With a positive coupling, the influence decays toward zero as disparity grows and remains an attraction, so that a repulsion of one estimate by the other, where it is observed, requires a negative coupling or a selection effect, since estimates conditioned on reports of separate sources can be repelled even under causal inference (Körding et al., 2007).

**Figure 2:**
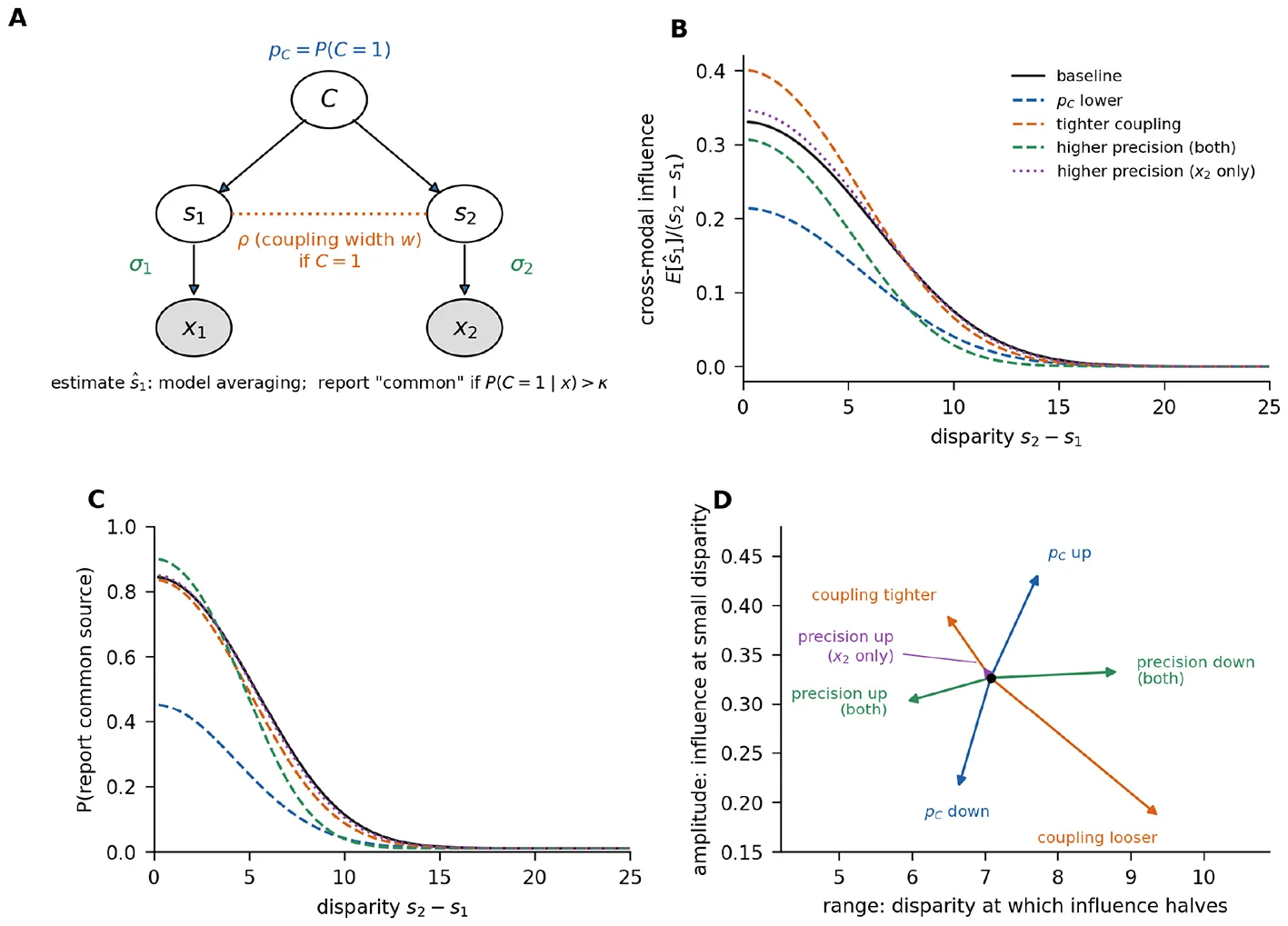
A generative model with two priors and its signatures. **(A)** The observer's model: a binary causal structure C with prior probability p_C_ of a common source; attribute values s_1_ and s_2_ that are correlated with coefficient ρ (coupling width w) when the source is common and independent otherwise; noisy measurements x_1_ and x_2_ with standard deviations σ_1_ and σ_2_. Estimates average the two causal structures, and a common source is reported when its posterior probability exceeds the criterion κ. **(B)** Cross-modal influence, the expected estimate of s_1_ divided by the disparity when s_1_ = 0, as a function of disparity for the baseline observer (p_C_ = 0.5, w = 2, σ_1_ = 3, σ_2_ = 1, σ_p_ = 10, illustrative units) and for changes of one component at a time: p_C_ = 0.3, w = 1, both noise levels reduced by 25%, and the noise of x_2_ alone reduced by 25%. **(C)** Probability of reporting a common source for the same observers. **(D)** Each change displaces the observer in the plane defined by the amplitude of the influence (at a disparity of one unit) and its range (the disparity at which it halves). Changes in p_C_ move the two in the same direction, and changes in the coupling, or in the precision of the influencing channel alone, move them in opposite directions; a joint change in precision narrows the range, whereas its effect on the amplitude depends on the coupling (Section 3.4).

### 3.5 A fixed common source

When p_C_ = 1, as with the color and odor of a wine in the glass, the model reduces to a bivariate Gaussian prior, and the estimate of odor becomes linear in the measurements of hue and odor. Consider a white wine, a rosé that differs from it along both attributes, and a white wine dyed to the hue of the rosé. The displacement of the dyed white toward the rosé, expressed as a fraction of the difference between the estimates for the rosé and for the white, has the closed form (Proposition 5)


$$\begin{array}{l} D=\frac{\rho \sigma_{o}^{2}}{\rho \sigma_{o}^{2}+\sigma_{p}^{2}(1-\rho^{2})+\sigma_{h}^{2}} \end{array}$$ (6)

where σ_h_ is the noise of the visual measurement of hue, σ_o_ the noise of the measurement of odor, and σ_p_ the spread of wines along each attribute, which is the same for both once they are expressed on a common scale, a choice of units that leaves their relation to ρ. The displacement increases with ρ, decreases as odor precision rises, and depends on the noise of hue only through its sum with $\sigma_{\text{p}}^{2}$(1 – ρ^2^), the variance of odor among wines of the same hue (Supplementary material S1 derives it). Unless the mapping from color to odor is close to deterministic, that variation is large compared with the visual noise in registering a hue, so that hue noise contributes little to the displacement, although a gain in hue precision raises displacement and differentiation together by a small amount (Figure 3B), so that both channel precisions must be measured before a joint increase is attributed to the coupling. The differentiation of the rosé from the white, measured by the discriminability of their odor estimates when both are seen, also increases with ρ over the range of parameters examined in Supplementary material S1. A stronger coupling thus produces, on its own, both features of the experienced tasters' profile reported by Wang and Spence (2019): with illustrative values, raising ρ from 0.3 to 0.8 raised the displacement from 0.25 to 0.69 and the discriminability d′ of rosé and white from 2.2 to 4.2, whereas a gain in odor precision alone raised d′ to 3.1 and lowered the displacement to 0.11 (Figure 3). Expression (6) also states in terms of cue combination what the coupling is in this case: the weight of color on the odor estimate is the reliability of color as a predictor of odor, which depends on the learned mapping as well as on the noise of hue, and on this account what distinguishes the experienced taster is the mapping. Since hue and odor are reduced to single dimensions on a common scale, expression (6) is a local linear approximation around the wines compared, and Figure 3 is qualitative with respect to the descriptor-profile measures of Wang and Spence (2019).

**Figure 3:**
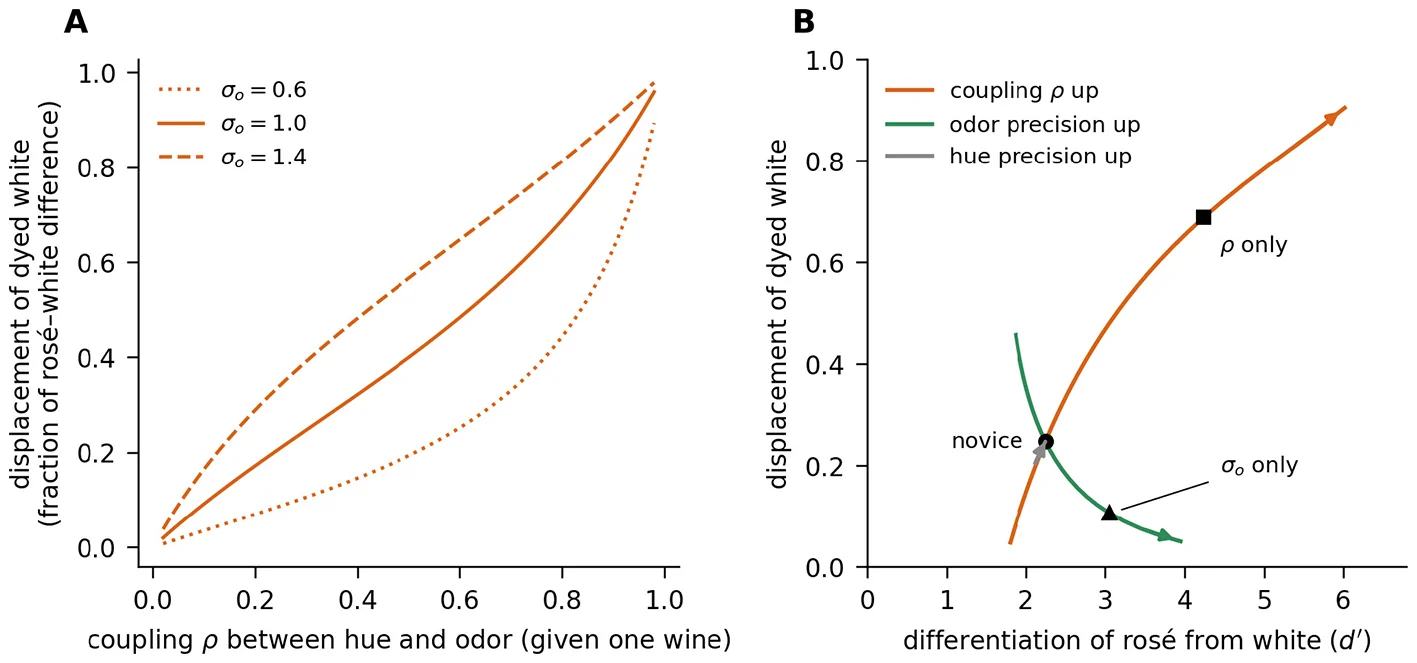
The chemosensory case with a fixed common source (p_C_ = 1). **(A)** Displacement of a white wine dyed to the hue of a rosé, as a fraction of the difference between rosé and white, as a function of the coupling ρ between hue and odor, for three levels of odor noise σ_o_ (expression 6; σ_p_ = 1, σ_h_ = 0.05). **(B)** Trajectories in the plane of differentiation (discriminability d′ of the odor estimates of rosé and white) and displacement: raising ρ from 0.05 to 0.95 (orange), raising odor precision (σ_o_ from 1.6 to 0.4 at ρ = 0.3, green), and raising hue precision (σ_h_ from 0.5 to 0.01, gray). Markers show a novice (ρ = 0.3, σ_o_ = 1), the same observer with ρ = 0.8, and the same observer with σ_o_ = 0.6. A tighter coupling increases displacement and differentiation together by a large amount (from ρ = 0.3 to 0.8, displacement from 0.25 to 0.69 and d′ from 2.25 to 4.24), and a gain in hue precision also increases both, by a small amount (along the gray trajectory, displacement from 0.21 to 0.25 and d′ from 2.12 to 2.25), so that both channel precisions must be measured before a joint increase is attributed to the coupling; a gain in odor precision increases differentiation while reducing displacement.

## 4 Environments, learning, and testable results

### 4.1 A proposal, and a classification rule

We propose that perceptual expertise adjusts channel precision, the prior of common cause, and the coupling prior separately, and that which of them moves is set by two statistics of the trained environment that vary independently of one another.

The first is the **base rate of common causation**: given two concurrent signals of the relevant kinds in this environment, how often do they in fact arise from a single source or event? The second is the **strength of the correlation**: given that they do arise from one source, how tightly does the value of one predict the value of the other? A high correlation is compatible with a low base rate, and a base rate of unity is compatible with any correlation.

Applied to the two cases, the rule classifies them cleanly and without appeal to the effects that it is meant to explain (Figure 1A). In ensemble music the correlation is very high, since instruments reliably make sound when played and the acoustic consequence of a gesture is nearly deterministic. The base rate of common causation, however, is low. A concert hall presents a great many concurrent visual and auditory events, and for an arbitrary pair of them the probability of a shared cause is small; the player's task is precisely to determine which visible gesture goes with which audible attack in a texture where most pairings are spurious. The correlation is therefore not a useful thing to learn, being already near-deterministic and no less available to the novice, and the useful adjustments are to raise channel precision, which improves the source-attribution judgment, and to lower p_C_, which prevents the readier fusion that would obscure the pairings that matter. This is the profile of Section 2.1.

In wine tasting, the base rate of common causation is unity. There is one glass, and neither expert nor novice entertains the hypothesis that the color they see and the odor they smell come from different objects, so p_C_ is not a free parameter for either group and cannot be what expertise adjusts. What is left to learn is the correlation, and there is a great deal to learn here, since hue is jointly determined with odor by grape variety, extraction, oxidative history, and age, among others, so that the mapping from fine gradations of color onto odor attributes is real, structured, and acquired only through sustained exposure. Expertise sharpens ρ, which is why the expert is displaced further when the experimenter breaks the correlation by dyeing the wine (Section 3.5). This is the profile of Section 2.2.

Two objections to this rule must be answered beforehand, since both bear on what “the environment” means. The first is that the base rate of common causation in an orchestra is a property of orchestras, and is therefore the same for the listener in the hall as for the player on the stage, so that it cannot explain a difference between them. The reply is that the relevant statistic is not the composition of the ambient scene but the distribution of inferential problems that the observer confronts, which is set by role and task rather than by what is physically present. The listener is not required to determine which visible gesture produced which audible attack and can leave the question unposed indefinitely; the player is required to solve it continuously and under time pressure, because coordination depends on it.

The second objection is that laboratory training regimes do not present rich scenes at all. McGovern et al. (2016) trained observers on a single flash and a single beep, a display in which the base rate of common causation is high, and yet their simulations could reproduce the data only with a reduced prior of common cause. The reply is that in the laboratory the inferential problem is imposed directly by instruction rather than mediated by scene statistics. An observer asked repeatedly to judge whether two signals were simultaneous is being required to treat the source question as live on every trial, which is what a low base rate does in the field. Scene statistics matter because, and to the extent that, they determine which questions an observer has to answer, and an experimenter can pose those questions without them. An explicit instruction about how often the signals were colocated lowered the fitted prior of common cause in musicians and non-musicians alike (O'Donohue et al., 2026), which is this direct route in its simplest form.

With those qualifications the rule is applicable in advance, whereas the descriptive taxonomies it replaces were not. To classify a domain, characterize the inferential problems its practitioners recurrently face, estimate the base rate of common causation and the correlation strength over those problems, and predict adjustment to whichever parameter the environment leaves free and informative. Three consequences follow immediately, one of which has already been tested.

First, audiovisual speech has both a high base rate of common causation, since one talker produces both streams, and a high correlation between articulation and acoustics. The rule therefore predicts that speech expertise should sharpen ρ and leave p_C_ alone, yielding more cross-modal capture. Second, the rule predicts that music training, which operates on p_C_ and on temporal precision, should not transfer to a task like the McGurk effect that assays the coupling between auditory and visual values in speech. That is the null that Lee et al. (2024) report, and which the field has treated as a failure to replicate an expertise effect. On our account, this successfully predicts domain-appropriate non-transfer, and we would not expect Proverbio et al. (2016) result to hold.

Third, the rule predicts that video-game expertise, acquired in audiovisually correlated environments that nonetheless present many concurrent independent events, should pattern with music rather than with wine, which is consistent with the sharpened multisensory temporal processing reported in action-game players (Donohue et al., 2010).

### 4.2 Why two parameters require different statistical information

Another reason to keep the two parameters separate is that separating them requires information beyond the covariance of the values, as the product result in Section 3.3 implies, although the result does not single out a biological learning mechanism.

A correlation between the values of two signals is exactly what a Hebbian or covariance-based rule operating on cross-modal connections will learn, since coincident activity in two populations strengthens the connection between them in proportion to how reliably it recurs. Circuit-level models and developmental findings in the superior colliculus illustrate this: manipulating the statistics of cross-modal experience during development changes the integrative profiles of the resulting neurons (Cuppini et al., 2012), and the multisensory transform in the superior colliculus is created by experience rather than specified in advance (Wang et al., 2020). Ernst's (2007) induction of a coupling prior after about an hour of training is compatible with such a rule.

A base rate of common causation is not learnable in this way on its own. It is a statistic over causal structures, carried by the higher-order shape of the joint distribution of values (Section 3.3), and estimating it requires the observer to represent the possibility that concurrent signals arose separately, which a covariance update does not do. What is required is an update that uses the higher-order shape of the joint distribution, such as the maximum-likelihood mixture learner of Supplementary Figure S2, which recovered both parameters where a covariance learner confounded them. Network models whose cross-modal synapses are trained by Hebbian potentiation acquire the statistics of the stimuli (Ursino et al., 2017), and networks of this kind reproduce the percept of unity and the pattern of bias as a function of disparity (Cuppini et al., 2017), which suggests that such learning stores more of the joint distribution than its correlation, leaving the base rate implicit in the connectivity until a decomposition like that of the mixture learner reads it out.

This is, we think, the substantive core of the proposal. The two literatures move different parameters because those parameters are carried by different features of the environment's statistics, and the appearance of contradiction was the appearance of one construct behaving inconsistently when in fact two constructs were behaving consistently.

### 4.3 Two results that appear to invalidate the account

McGovern et al. (2016) trained observers on audiovisual temporal discrimination and found a narrowing of the temporal binding window specific to the trained dimension, along with a general reduction in cross-modal interaction that their model attributed to a decrease in the prior expectation of a common source. Read as a general statement about expertise, this contradicts any claim that training raises a prior. Read through the distinction we propose, it does not, however, since the training regime was temporal discrimination, which is the source-attribution regime in which our rule predicts that p_C_ should fall while precision rises. The dissociation that they report between temporal and spatial transfer is what a parameter-specific account expects.

Two further results challenge our view. Hong et al. (2022) found that p_C_ changed during learning for most participants while the direction of change was unreliable at the individual level, with roughly half updating against the statistics of their exposure. This is evidence against a simple statistical-learning account of p_C_, and our proposal requires such an account. We regard it as an open problem; Li et al. (2025), from the same laboratory, model temporal recalibration under a fixed common-cause prior, so that whether the prior itself recalibrates remains untested. Separately, Odegaard and Shams (2016) showed that p_C_ is stable within individuals but does not generalize across a spatial and a temporal audiovisual task, though those authors leave open whether the failure reflects task specificity or domain specificity. Either way, non-transfer is not, by itself, a signature of expertise, and our account needs the expert-vs.-novice contrast within a domain.

### 4.4 The single-parameter rival

The most parsimonious objection to this study is that the musician profile requires no second parameter at all. Parise and Ernst (2016) show that a multisensory correlation detector reproduces the temporal binding window, temporal order and simultaneity judgments, and Bayes-optimal localization from the time constants of its filters and a divisive normalization stage, without any parameter corresponding to p_C_. Shorter filter constants in musicians would yield narrower windows, reduced cross-modal capture, and better unimodal timing, which is most of Section 2.1. Within the model of Section 3, precision alone narrows the window in every regime, and it reduces capture at small disparities only when the coupling is loose (Section 3.4), whereas redundant attributes such as the timing of a single event are tightly coupled.

The musician evidence alone does not require two parameters, although the causal-inference fit of O'Donohue et al. (2026) locates the reduced integration of musicians in the common-cause prior with precision matched, which is the parameter that the classification rule assigns to them. The second parameter is the conjunction with taste evidence, specifically the pattern identified at the end of Section 2.2, in which experienced groups discriminated wines that differed in composition more effectively while being less affected by color. Within the model, a gain in odor precision improves discrimination and reduces displacement, whereas tighter coupling produces both (Proposition 5, the closed form for the displacement), so that no change in the reliability of the chemosensory channel yields the pattern. The correlation-detector account, which has no prior over the values of an arbitrary learned mapping, has still less purchase here, since the color-odor correspondence is not a temporal correlation between stimulus envelopes.

A fair statement of our position is therefore that the second parameter earns its place on the chemosensory side and is, on the musician side, a hypothesis that the design of Section 8.1 is built to test. Distinguishing a change in filter time constants from a change in p_C_ requires fitting both model classes to the same data across a disparity range, a route that to our knowledge has not been pursued so far by any study in an expert population, and distinguishing a change in precision from a change in p_C_ requires measuring precision independently, which Section 7 shows to be sufficient in a battery that includes large disparities.

## 5 The chemosensory case reconsidered

### 5.1 Bias or calibration

The wine literature describes color-induced effects in the vocabulary of illusion, bias, error, and deception. We propose that this vocabulary misdescribes what has been demonstrated, though the argument has two parts, because the two studies at issue do not support the same conclusion.

The case of Morrot et al. (2001) is the weaker one, weaker even than the reinterpretation usually offered for it. In the critical session, participants received two glasses, one containing white wine and another the same white wine dyed red with an odorless preparation, and were required to assign each descriptor from their own previously generated list to whichever glass presented that character more intensely. The two samples were identical in every respect bearing on odor, so the olfactory channel carried no information about the required discrimination and the posterior over that discrimination was flat. Under a flat posterior, every allocation strategy is equally consistent with the evidence, including responding at chance. What follows is therefore not that color-based allocation was uniquely correct, but that the design cannot distinguish a perceptual displacement from a rational tie-break, and that the paper's conclusion that “the tasters discounted the olfactory information” attributes a failure to observers who had no olfactory information to discount. The study therefore does not demonstrate the phenomenon it is cited for.

The case of Wang and Spence (2019) differs because the rosé and the dyed white were different wines and therefore differed in odor, even though the descriptions participants produced tracked color. Here the displacement is real, and consequently the reinterpretation has force. Dyeing a white wine to match a rosé breaks a correlation that holds robustly outside the laboratory. An observer whose prior encodes that correlation will be displaced in proportion to how tightly the prior is specified, as expression (6) makes explicit. Thus, the observer whose prior is best calibrated to the natural environment is displaced furthest. Greater susceptibility in the experienced groups is, on this reading, a signature of better learning, and the appropriate description is that the experiment was constructed to exploit what experts know.

Two limits on this argument must be stated. First, displacement is governed jointly by the coupling and by the precision of the odor channel, as expression (6) shows, so that it is diagnostic of ρ only when odor precision is matched across the groups compared. The observation that studies have tended to document more pronounced changes in conceptual structure than in chemosensory sensitivity (Spence and Wang, 2019), a pattern that those authors themselves attribute in part to the limitations of threshold testing, makes the matching assumption defensible without establishing it. Second, and more seriously, the strength of the expert prior is currently inferred from the displacement it is invoked to explain. The argument is therefore not falsifiable until an independent estimate of ρ is available, which is the point of the design proposed in Section 8.2.

### 5.2 A perceptual or a decisional locus

The most serious alternative holds that the expert advantage in the wine case is not perceptual at all, but resides in a richer descriptive vocabulary, better-organized semantic knowledge, and more effective retrieval. On this reading, the displaced expert has not integrated color into a perceptual estimate but has selected descriptors from a lexicon in which color terms and odor terms are already associated, so that the effect lives at the level of verbal report.

In terms of Section 3, the two readings place the coupling in different parts of the observer. On the perceptual reading, ρ belongs to the prior over values, and the estimate of odor is itself displaced toward what the color predicts. On the decisional reading, the estimate is unchanged, and the displacement arises in the mapping from estimates to reports; in a descriptor task, it is a choice among verbal labels whose prior probabilities depend on the color seen, so that color acts on the response criterion. In a categorical report, a shift of the internal estimate and a shift of the criteria or label priors that map estimates onto labels can produce the same distribution of responses, as in signal detection theory, so that the two readings predict the same descriptor profiles; Proposition 3 states the analogous result for reports of a common source. They diverge when responses no longer pass through the lexicon, since a decisional coupling should vanish when responses are continuous and non-verbal, whereas a perceptual coupling should persist.

This explanation cannot be dismissed on present evidence, and one of its principal sources of support comes from authors sympathetic to the perceptual reading, since Spence and Wang (2019) conclude that wine expertise produces more pronounced changes in conceptual knowledge structures than in smell or taste sensitivity as such, and that differences at the level of conceptual knowledge are far more readily apparent than differences in chemosensory acuity. Morrot et al. (2001) themselves locate the effect at the verbalization phase of odor determination. The dependent measures in every study reviewed in Section 2.2 are descriptor profiles and verbal identifications, which is where a semantic account predicts the effect to appear.

The response modality, not the model fit, is therefore the discriminating factor, since a semantically driven response bias would still register as an elevated fitted parameter if the response remained verbal, and the non-verbal task of Section 8.2 provides that modality. Croijmans et al. (2020) add a complication that we record as an issue for our position. They found that wine expertise raises the reported vividness of wine imagery across color, odor, and taste, and that the increase followed a 6-month course of theory and tasting rather than tracking declarative wine knowledge. They state explicitly, however, that their design does not distinguish perceptual sharpening from enriched semantic and verbal association, and their dependent measure is self-reported imagery rather than perceptual performance. The same group earlier found that wine and coffee experts were no more accurate than novices at identifying everyday smells and tastes; their advantage lay in source-specificity of description and, for wine experts specifically, in its consistency (Croijmans and Majid, 2016). This is the shape of evidence the semantic account predicts, although a later study found that linguistic measures of expert descriptions did not predict the vividness of expert imagery, so that imagery and language appear to be partly separate facets of wine expertise (Croijmans et al., 2024).

We regard the question as open and would not claim that the perceptual reading is established. It bears noting that the semantic account has no purchase on the musician results, which involve no descriptive vocabulary, so on the semantic reading the two literatures are simply unrelated rather than contradictory, which is a coherent position and one that our account must outperform.

## 6 Implementation

### 6.1 Candidate substrates

A two-parameter account requires that the parameters be separately implementable, and the multisensory hierarchy offers one candidate architecture. Rohe and Noppeney (2015a, 2016) showed that the human brain computes the several estimates that a causal-inference model posits at distinct cortical levels, with segregated unisensory estimates in early sensory areas, a forced-fusion estimate in the posterior intraparietal sulcus, and the model-averaged estimate that reflects the causal-inference computation proper in the anterior intraparietal sulcus. Cao et al. (2019) report a comparable cascade, though they place the causal-inference computation in the prefrontal cortex, so that the two lines of work converge on hierarchical organization while differing on locus.

The two priors need not map onto successive stages of this hierarchy, and the assignment that the architecture invites, with the coupling prior in the forced-fusion computation and the common-cause prior at the arbitration stage, is one possibility among several. In the model of Section 3, the coupling is a property of the prior over values given a common source, and a natural candidate for its substrate is the learned connectivity between the populations that encode the two values, which correlational plasticity shapes during development and training (Cuppini et al., 2012; Ursino et al., 2017; Wang et al., 2020). The common-cause prior is a property of the inference over causal structures, for which several implementations have been proposed: populations of congruent and opposite neurons, whose balance of activity supports integration and segregation concurrently (Zhang et al., 2019) and which emerge in networks trained to solve causal inference (Rideaux et al., 2021), and sampling-based codes, in which posterior probabilities are expressed in the frequency with which neural activity visits each hypothesis (Fiser et al., 2010). The prediction is that expertise should dissociate the two, with musicians showing a shift toward segregation in the read-out that arbitrates between causal structures and tasters showing sharpened cross-modal weights in the estimate of values, and neither showing the other. Figure 1B summarizes these candidates, whose loci remain open.

For channel precision the substrate is better established. Divisive normalization accounts for a wide range of multisensory response properties in the cortex (Ohshiro et al., 2011, 2017), and in a probabilistic population code reliability is expressed as population gain (Ma et al., 2006), which is the identity that makes precision a physical rather than a purely formal quantity. Fast adjustments of that gain are under neuromodulatory control, cholinergic signaling in particular having been characterized as reporting expected uncertainty (Yu and Dayan, 2005) and, within the free-energy formulation, as setting the precision of prediction errors (Moran et al., 2013).

### 6.2 Timescales

The three parameters differ in how quickly they move, and the distinction is worth making explicit because it constrains the designs of Section 8. Channel precision is adjusted within and across trials by attention and neuromodulation. The coupling prior is learned over hours in the laboratory (Ernst, 2007; Negen et al., 2018) and presumably over years in the field. On the evidence of Section 4.3, the prior of common cause is plastic under training and unreliable in its direction, which is consistent with a slower, more weakly constrained structure-learning process.

## 7 Recoverability of the two priors

A dissociation between the two priors can be tested only if the parameters that it concerns can be recovered from data of realistic size. We therefore simulated a test battery designed for the purpose and fitted the model of Section 3 to the simulated data (Supplementary material S2). Each session combined three kinds of trials. On bimodal trials, the two signals were presented at one of five positions and with one of nine disparities, up to ±18 units, a range that spans the transition from integration to segregation at the parameter values considered, and the observer reported both a continuous estimate of s_1_ and whether the two signals shared a source. In 100 unimodal estimation trials, each signal was presented alone, and its value was reported, which fixes the width of the prior over values through the central tendency of the reports. On 240 unimodal discrimination trials, the observer compared two presentations of the same channel, and the psychometric slope measured the precision of each channel independently of the noise of report. All seven free parameters, including the report noise σ_m_ and the criterion κ, were estimated jointly by maximum likelihood, with the estimate and the report of each bimodal trial entering one likelihood (numerical details in Supplementary material S2), and the generating values of the 30 simulated observers at each trial count were drawn from wide ranges (Supplementary material S2).

With 900 bimodal trials, the correlation between generating and recovered values was 0.94 for logit p_C_, 0.89 for the coupling width, 0.97 and 0.90 for the two noise levels and 0.86 for the criterion, whereas the prior width and the report noise were recovered less well (0.67 and 0.70; Figures 4A–C and Supplementary Figure S3). The recovery of the coupling width depended on its size relative to sensory noise. When w amounted to at least half of the combined noise ($\sigma_{1}^{2}$ + $\sigma_{2}^{2}$)^1/2^, its mean relative error was 13%; below that level the error rose to 66%, and 6 of the 17 fits in that region placed the coupling at the lower bound of the fitting range, close to the limit of causal inference. This boundary follows from Proposition 4: the coupling enters the responses through V_1_ = w^2^ + $\sigma_{1}^{2}$ + $\sigma_{2}^{2}$, and a coupling that is small relative to sensory noise changes V_1_ by less than the uncertainty with which the noise itself is estimated. Recovery improved with the number of trials, the correlation for w rising from 0.56 with 225 bimodal trials to 0.92 with 1,800, while the estimation errors of p_C_ and w were moderately correlated (0.41 with 900 trials), as expected from their joint effect on the amplitude of the influence.

**Figure 4:**
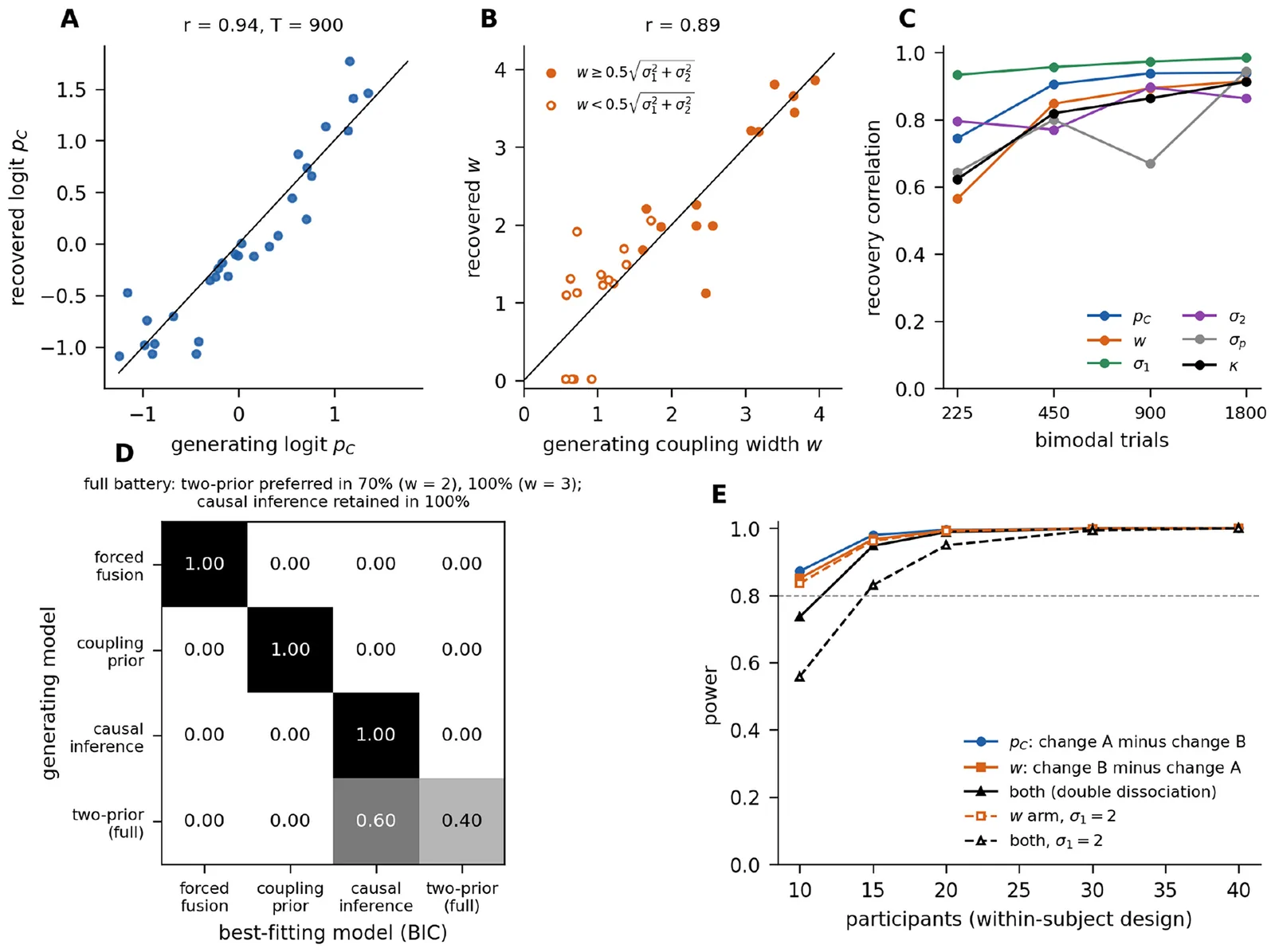
Recoverability of the two priors in the test battery of Section 7. **(A, B)** Generating and recovered values of logit p_C_ and of the coupling width w for 30 simulated observers with 900 bimodal trials; open symbols mark couplings smaller than half of the combined sensory noise. **(C)** Correlation between generating and recovered values as a function of the number of bimodal trials. **(D)** Model recovery on continuous estimates and unimodal data: proportion of datasets generated by each model (rows) for which each model (columns) had the lowest Bayesian information criterion (BIC), with 20 datasets per generating model. With the full battery, including common-source reports, the two-prior model was preferred to causal inference in 70% of datasets generated with w = 2 and 100% with w = 3, and causal inference was retained in 100% of datasets generated without coupling. **(E)** Power of the within-subject design for the contrast between the changes in p_C_ produced by the two protocols, the corresponding contrast for w, and both, as a function of the number of participants (4,000 simulated experiments per point; illustrative effect sizes, Supplementary material S2); dashed lines show the same design with a more precise target channel (σ_1_ = 2 before training).

Model recovery was assessed in two steps. When the two-prior model and its three special cases (Proposition 1) were fitted to the continuous estimates and the unimodal data alone, forced fusion, the coupling-prior model and causal inference were each identified in all 20 datasets that they generated, whereas data generated by the two-prior model with a coupling width of 2 units were attributed to causal inference in 60% of cases (Figure 4D). Continuous estimates therefore indicate whether a causal structure is inferred, but they provide a weak basis for detecting a coupling of this size against the zero coupling of causal inference. The common-source reports provide much of the missing evidence, since the rate at which they fall with disparity depends on V_1_ (Proposition 4). With the full battery, the two-prior model was preferred to causal inference in 70% of the datasets generated with w = 2 units and in 100% of those generated with w = 3 units, while causal inference was retained in 100% of the datasets generated without coupling, so that the comparison errs, where it does, toward the simpler model (Wilson 95% intervals in Supplementary material S2).

Each component of the battery contributes to identification, as the reduced designs show (Supplementary Figure S1; the values are given in Supplementary material S2). With common-source reports alone, the likelihood was exactly flat along the line on which logit p_C_ – logit κ is constant, as Proposition 3 requires, and the continuous estimates restored its curvature. Without common-source reports, p_C_ remained identified by the estimates, although less precisely; without unimodal trials for the influencing channel, the coupling width and the noise of that channel traded against each other; precision measured only through the variability of unimodal estimates, which mixes sensory noise with the noise of report, was underestimated, and the coupling width absorbed the difference; and disparities limited to ±5 units left both priors estimable but raised the correlation between their estimates.

The battery also bears on the difficulty raised in Section 2.1 for the musician evidence. When only the precision of both channels increased, with p_C_ unchanged, fits that included the unimodal trials left the estimate of p_C_ in place (mean change in logit p_C_ of +0.01). Without the unimodal trials, the mean change remained small (−0.11), since the estimates at large disparities, where the signals are segregated, depend on the target channel alone and reveal its noise, whereas the standard deviation of the estimate rose to 0.47 against 0.27 and the noise of the influencing channel was underestimated together with the coupling width. Designs that rely on binary judgments and do not measure unimodal precision lack both of the elements that make this separation possible.

Finally, we estimated the power of a within-subject test of the double dissociation, in which each participant is tested with the full battery before and after each of two training protocols. Protocol A, which rewards the detection of discrepancies between the two channels, was assumed to lower p_C_ from 0.5 to 0.3 and to reduce the sensory noise of both channels by 15%; protocol B, which rewards the inference of a hidden property from two cues held in reliable relation on a single object, was assumed to halve the coupling width while leaving p_C_ unchanged. The variability of the changes across participants and the resampling of estimation errors are described in Supplementary material S2. With 900 bimodal trials per session, the contrast between the changes in p_C_ produced by the two protocols reached a power of 0.80 with 10 participants; the corresponding contrast for w with 10, and both contrasts together with 15, the smallest samples of the simulated grid at which power reached 0.80 (Figure 4E); a non-central-*t* approximation reproduced these values within 0.011. The coupling arm is the more fragile of the two. After protocol B, the coupling width lay at about a third of the combined sensory noise; 6 of the 30 fits placed it at the lower bound of the fitting range, and the resulting bias (−0.12 units) inflates the detected decrease. A more precise target channel did not remove this difficulty (Supplementary material S2). The predicted dissociation was also recovered when simulated observers matched probabilities while the fitted model averaged, with power of 0.80 for both effects at 10 to 15 participants in two sets of simulations, although the fitted coupling then widened after protocol A by a third to a half of its true narrowing after protocol B and was strongly biased in absolute terms, about half its true value before training and at the lower bound after protocol B; it was not recovered when they selected the more probable structure, in which case the fitted coupling absorbed the changes produced by protocol A and missed the narrowing produced by protocol B (Supplementary material S2), so that the read-out of each observer must be established by comparing fits before parameters are compared across sessions. Power also depends on the finite library of fitting errors on which it draws: resampling that library gives power for both contrasts with 15 participants between 0.82 and 0.97 for 95% of libraries (0.56 to 0.94 with a more precise target channel), whereas a bounded spread of the coupling changes leaves it essentially unchanged (Supplementary material S3). These values rely on illustrative effect sizes and indicate that the test is feasible with conventional sample size, whereas a power analysis for a specific study requires effect sizes estimated from pilot data.

## 8 Predictions and empirical program

We order the predictions by how cleanly each discriminates the account, and for each state the manipulation, the measure, and the result that would falsify our account, as Table 2 summarizes.

**Table 2:** Predictions, manipulations, measures, and results that would count against the account.

Prediction	Manipulation or comparison	Measure	Predicted result	Result counting against the account
Protocol-by-parameter dissociation (Section 8.1)	Two training protocols within subjects: detection of discrepancy (A); inference of a hidden property from two correlated cues on one object (B)	p_C_, w, precisions and κ fitted to the battery of Section 7	A lowers p_C_; B narrows w; neither moves the other	No interaction, or both protocols moving the same parameter
Signatures within one dataset (Sections 3.4 and 8.2)	Disparity sampled across the transition from integration to segregation	Amplitude and range of cross-modal influence, with precision measured by discrimination	p_C_ moves amplitude and range together; the coupling moves them apart	Changes that match neither pattern once precision is accounted for
Chemosensory coupling (Sections 3.5 and 8.2)	Experts and novices on a graded hue-by-aroma continuum	Continuous non-verbal odor estimates; differentiation of congruent wines	Experts show more displacement and better differentiation at matched odor precision	More displacement without better differentiation, or an effect confined to verbal responses
Expert populations (Section 8.3)	Sommeliers and trained musicians, each tested in both domains against novices	p_C_ and ρ fitted within each domain	Lower p_C_ in musicians for temporal tasks; higher ρ in sommeliers for hue and odor	Parameter differences absent within domain or opposite in sign
Domain-appropriate non-transfer (Section 4.1)	Musical training against audiovisual coupling in speech	McGurk fusion with graded measures of training	No relation with musical training	Reduced fusion in musicians with graded measures of training

### 8.1 Opposite parameter movement under two training protocols

Within a single population and using common test stimuli, two protocols should move different parameters. A protocol rewarding detection of temporal discrepancy should narrow the window and lower the fitted p_C_, replicating McGovern et al. (2016). A protocol rewarding inference of a hidden property from two cues held in reliable correlation, with the base rate of common causation fixed at unity by presenting a single object, should raise fitted ρ, narrowing the coupling width, while leaving p_C_ unchanged. The critical result is a protocol-by-parameter interaction in the predicted directions, and the account is refuted by its absence.

Three design requirements follow from Section 7 and from the training literature. A yoked control must match disparity exposure and feedback rate across protocols, since the protocols otherwise differ in difficulty and conflict exposure. The test battery must combine continuous estimates with common-source reports across disparities that reach the region of segregation, and it must measure the precision of each channel by discrimination and identify the read-out of each observer, since each of these components carries part of the identification. Furthermore, the coupling after training should remain at least half the size of the combined sensory noise; below that threshold, it cannot be distinguished from zero coupling in causal inference. Section 7 indicates that, with these requirements met, the dissociation is detectable with conventional sample sizes (Figure 4E). Protocol order must be counterbalanced with a washout between protocols, since the power analysis assumes no carryover between protocols and each test session comprises about 1,240 trials. We regard this as the most economical test of the two-parameter claim: it is within-subjects, requires no expert recruitment, and Negen et al. (2018) suggest that 2 h of training may suffice to induce reweighting of a newly learned cue.

We note that the second arm resembles (Ernst, 2007), which induced a coupling prior by training a correlated mapping. What is new is the within-subject contrast against the first arm and the joint fitting of both parameters, which Ernst did not attempt.

A recent preprint reports a dissociation of the kind that the first arm targets: after simultaneity-judgment training that produced the same behavioral gain in both groups, the fitted common-cause prior fell in typically developing children, whereas children with attention-deficit/hyperactivity disorder (ADHD) showed reduced temporal uncertainty without a change in the prior (Zhu et al., 2026).

### 8.2 Signatures within a single dataset

The cheapest discrimination of the parameters within a single dataset uses the shape of the cross-modal influence across disparity, whose signatures are derived in Section 3.4 (Figure 2); in the chemosensory case, they connect to the assimilation-and-contrast structure of the food expectation literature (Piqueras-Fiszman and Spence, 2015), whose contrast regime would require the extensions noted in Section 3.4.

Making this measurable in wine requires specifying an axis, which no existing study does to our knowledge. We propose a graded hue continuum from white through rosé to red crossed with a graded aroma continuum, with responses on a trained perceptual scale rather than in free description, which also addresses the decisional alternative of Section 5.2 by removing the descriptive lexicon from the response.

### 8.3 Coupling prior and p_*C*_ in real expert populations

Estimates of p_C_ are routine in psychophysics, and the first estimate in an expert population has shown a lower prior in musicians (O'Donohue et al., 2026), whereas the two studies estimating change under learning used short laboratory training in naive participants (McGovern et al., 2016; Hong et al., 2022). Estimates of the coupling in an expert population are, to our knowledge, still lacking, and fitting the model of Section 3 to sommeliers and to professionally trained musicians, each tested in both domains, would provide them.

Landry et al. (2017) suggest that what transfers is tied to the type of inferential problem, which implies that the control should cross problem type with modality pair: musicians tested on a chemosensory temporal task should show the effect, and on a non-temporal audiovisual task should not.

## 9 Discussion

### 9.1 Limitations

#### 9.1.1 The evidential base for the amplification effect

Section 2.2 rests substantially on one study that reports its central comparison descriptively, classifies expertise by self-report, and has a novice group confounded by a discriminability floor, with two further studies finding no expertise effect. If the amplification effect proves null, which the reanalysis of Supplementary material S4 does not exclude, the framework reduces to a claim about the constructed environments of Section 8.1, which remains testable but loses its motivating case.

#### 9.1.2 Identifiability

The simulations of Section 7 establish that the two priors, the precisions and the criterion, can be recovered from a battery of realistic size, and they locate the conditions under which recovery fails: without continuous estimates the common-cause prior cannot be separated from the criterion, without independent measures of precision the coupling cannot be separated from the noise of the influencing channel, and couplings that are small relative to sensory noise cannot be told apart from zero. These results concern simulated observers that follow the model, and whether human observers do so with parameters in the range explored is an empirical question.

#### 9.1.3 Ballester and colleagues

Their finding that novices categorize wines by color class from odor as accurately as experts is not accommodated by an account in which experts have learned a sharper color-odor mapping, and we do not have an explanation for it.

#### 9.1.4 Selection

We have treated expertise as the product of training. Training studies show that these parameters are plastic, indicating that selection is not the whole story and does not exclude its contribution in real expert populations.

#### 9.1.5 The formal treatment

The model assumes Gaussian priors and noise, equal marginal distributions under the two causal structures, and model averaging as the read-out. The criterion confound of Section 3.4 does not depend on these assumptions and the product result requires only the second, whereas the quantitative signatures and the recovery results do depend on them, and a fit that assumes model averaging misreads observers who select a causal structure (Section 7).

### 9.2 Conclusions

The literature on perceptual expertise and multisensory processing shows two patterns pointing in opposite directions, one well documented and one probable but contested. We have stated a generative model in which the two are separate parameters, shown that the familiar correlational statistic of an environment carries only their product, and derived signatures and a test battery through which they can be separated. We have argued that they are not one parameter behaving inconsistently but two parameters behaving consistently, and that the field's habit of naming both the coupling prior and the prior of common cause with a single phrase has concealed the distinction. The ensemble musician learns in an environment of high correlation and low base rate of common causation and adjusts precision and the prior of common cause; the taster learns in an environment where the base rate is fixed at unity, and only the correlation is free and adjusts the coupling prior. Because the two statistics vary independently, the account classifies environments it has not seen, and because the two parameters are carried by different features of the environment's statistics and require different information to be learned, the distinction has a mechanistic basis as well as a formal one.

Three lines of work follow. The two-protocol training study of Section 8.1 tests the central dissociation within subjects and would either establish it or remove the framework's foundation. Fitting the model to real expert populations would yield parameter estimates that currently exist only for the common-cause prior in musicians (O'Donohue et al., 2026). Moreover, the signatures of Section 3.4 offer a within-dataset discrimination of the two parameters that requires no new populations at all.

Beyond the framework, the account offers a reinterpretation of results that have been read as a catalog of expert failure. When an experienced taster describes a dyed white wine in the vocabulary of rosé, the taster applies a prior that encodes a correlation holding robustly outside the laboratory, which the experimenter has deliberately broken. If an independently estimated prior is elevated among experts, the displacement measures how well that correlation has been learned. Reading it as a failure of discrimination will mistake the calibration of a perceptual system for a malfunction.

## Data Availability

The original contributions presented in the study are included in the article/Supplementary material, further inquiries can be directed to the corresponding author.
