# Supplementary material for: Resolving the apparent paradox of multisensory expertise: coupling priors, common-cause priors, and how to distinguish among them
Source: Front Hum Neurosci. 2026 Oct 29;20:1960361. doi: 10.3389/fnhum.2026.1960361 (PMC13754695; doi:10.3389/fnhum.2026.1960361)
Supplement: Supplementary file 1 [file Supplementary_File_1.docx]

**Supplementary Material**

*Resolving the apparent paradox of multisensory expertise: coupling priors, common-cause priors, and how to sort them out*

Alessandro Bortolotti, Riccardo Palumbo, Fabio Lokwani-Di Matteo and Pier Luigi Sacco

# Supplementary Material S1: Propositions and proofs

*The model.* A binary variable $C$ indicates whether two signals share a source ($C=1$) or arise from independent sources ($C=2$), with $P\left( C=1 \right)=p_{C}$. Given $C=c$, the attribute values $\mathbf{s}=\left( s_{1},s_{2} \right)$ are Gaussian with mean zero and covariance $\Sigma_{c}$, where $\Sigma_{1}=\sigma_{p}^{2}\left( \begin{matrix} 1 & \rho\\ \rho& 1 \end{matrix} \right)$ and $\Sigma_{2}=\sigma_{p}^{2}I$, and the measurements are $\mathbf{x}=\mathbf{s}+\boldsymbol{\varepsilon}$ with $\boldsymbol{\varepsilon}\mathcal{\sim N}\left( \mathbf{0},R \right)$ and $R=diag\left( \sigma_{1}^{2},\sigma_{2}^{2} \right)$, so that the coupling $\rho$ is a correlation between the values and the measurement noise is independent across the two channels. We write $A_{c}=\Sigma_{c}+R$, and $w^{2}=2\sigma_{p}^{2}\left( 1-\rho\right)$ for the squared coupling width. The observer's log posterior odds of a common source are $L\left( \mathbf{x} \right)=logitp_{C}+log\mathcal{N}\left( \mathbf{x};\mathbf{0},A_{1} \right)-log\mathcal{N}\left( \mathbf{x};\mathbf{0},A_{2} \right)$, with $\mathrm{logit}p=log\left[ p/\left( 1-p \right) \right]$, and $P\left( C=1\mid\mathbf{x} \right)=1/\left[ 1+exp\left( -L\left( \mathbf{x} \right) \right) \right]$; its estimate of the values is the model average $\hat{\mathbf{s}}=P\left( C=1\mid\mathbf{x} \right) \Sigma_{1}A_{1}^{-1}\mathbf{x}+\left[ 1-P\left( C=1\mid\mathbf{x} \right) \right] \Sigma_{2}A_{2}^{-1}\mathbf{x}$; and a common source is reported when $P\left( C=1\mid\mathbf{x} \right)$ exceeds a criterion $\kappa$. These relations are equations (1) to (3) of the main text. All symbolic steps were verified with sympy, and every closed form was compared with an independent numerical computation (script derivations.py and the verification scripts in the replication package).

**Proposition 1 (nesting).** (a) For $\rho=1$, $\mathcal{N}\left( \mathbf{x};\mathbf{0},A_{1} \right)$ equals the likelihood of a common cause in Körding et al. (2007), and the model-averaging estimates equal theirs. (b) For $p_{C}=1$, the estimate is $\Sigma_{1}A_{1}^{-1}\mathbf{x}$, the posterior mean under a single bivariate Gaussian (coupling) prior. (c) For $p_{C}=1$ and $\rho=1$, the model is forced fusion with a Gaussian prior.

*Proof.* (a) With $\rho=1$, $\Sigma_{1}=\sigma_{p}^{2}\mathbf{1}\mathbf{1}^{\top}$, so that $s_{1}=s_{2}=s\mathcal{\sim N}\left( 0,\sigma_{p}^{2} \right)$ under a common cause and $\mathbf{x}\mid C=1\mathcal{\sim N}\left( \mathbf{0},\sigma_{p}^{2}\mathbf{1}\mathbf{1}^{\top}+R \right)$. Its determinant is $\sigma_{1}^{2}\sigma_{2}^{2}+\sigma_{1}^{2}\sigma_{p}^{2}+\sigma_{2}^{2}\sigma_{p}^{2}$ and its quadratic form is $\left[ \left( x_{1}-x_{2} \right)^{2}\sigma_{p}^{2}+x_{1}^{2}\sigma_{2}^{2}+x_{2}^{2}\sigma_{1}^{2} \right]/\left( \sigma_{1}^{2}\sigma_{2}^{2}+\sigma_{1}^{2}\sigma_{p}^{2}+\sigma_{2}^{2}\sigma_{p}^{2} \right)$, which is the expression of Körding et al. (2007) with zero prior mean. The first component of $\Sigma_{1}A_{1}^{-1}\mathbf{x}$ reduces to $\left( x_{1}/\sigma_{1}^{2}+x_{2}/\sigma_{2}^{2} \right)/\left( 1/\sigma_{1}^{2}+1/\sigma_{2}^{2}+1/\sigma_{p}^{2} \right)$, and under independent causes to $\left( x_{1}/\sigma_{1}^{2} \right)/\left( 1/\sigma_{1}^{2}+1/\sigma_{p}^{2} \right)$, which are their estimates. (b) and (c) follow because $P\left( C=1\mid\mathbf{x} \right)=1$ when $p_{C}=1$. $▫$

*Remark (the Gaussian coupling prior as a limit).* In the coordinates $u=\left( s_{1}+s_{2} \right)/\sqrt{2}$ and $v=\left( s_{2}-s_{1} \right)/\sqrt{2}$, the common-source prior has variance $\sigma_{p}^{2}\left( 1+\rho\right)$ along the identity line and $\sigma_{p}^{2}\left( 1-\rho\right)=w^{2}/2$ across it, with no covariance between the two. Letting $\sigma_{p}\to\infty$ with $w$ fixed, so that $\rho\to1$, the variance along the identity line diverges while the cross-section keeps the variance $w^{2}/2$: the prior becomes a Gaussian ridge of width $w$ that is flat along the identity line, which is the coupling prior of Ernst (2005, 2007) and Bresciani et al. (2006), and the estimate $\Sigma_{1}A_{1}^{-1}\mathbf{x}$ converges to the posterior mean under that prior. For finite $\sigma_{p}$ the two priors differ only in the finite variance along the identity line, which shrinks the common component of the two estimates, their component along that line, toward the prior mean.

**Proposition 2 (second-order statistics).** (a) $\mathrm{Corr}\left( s_{1},s_{2} \right)=p_{C} \rho$, and the slope of the regression of either value on the other equals $p_{C} \rho$. (b) The difference $d=s_{2}-s_{1}$ has excess kurtosis $\gamma_{2}=3p_{C}\left( 1-p_{C} \right)\rho^{2}/\left( 1-p_{C}\rho\right)^{2}$, which is zero if and only if $p_{C}\in\{0,1\}$ or $\rho=0$ (when $p_{C}=1$ and $\rho=1$ the difference is identically zero and its kurtosis is undefined). (c) Given $c=p_{C}\rho\in\left( 0,1 \right)$ and $\gamma_{2}$, $p_{C}=3c^{2}/\left( 3c^{2}+\gamma_{2}\left( 1-c \right)^{2} \right)$. (d) A delta-rule learner $b_{t+1}=b_{t}+\eta_{t}\left( s_{2,t}-b_{t}s_{1,t} \right)s_{1,t}$ with Robbins-Monro step sizes converges to $p_{C}\rho$.

*Proof.* (a) Both components have zero means and marginal variances $\sigma_{p}^{2}$, so $\mathrm{Var}\left( s_{i} \right)=\sigma_{p}^{2}$ and $\mathrm{Cov}\left( s_{1},s_{2} \right)=p_{C}\rho\sigma_{p}^{2}+\left( 1-p_{C} \right)\cdot0$. The regression slope is $Cov/Var$. (b) Conditionally on $C$, $d$ is Gaussian with variance $v_{1}=2\sigma_{p}^{2}\left( 1-\rho\right)$ or $v_{2}=2\sigma_{p}^{2}$. For a scale mixture, $E\left[ d^{4} \right]=3\left( p_{C}v_{1}^{2}+\left( 1-p_{C} \right)v_{2}^{2} \right)$ and $E\left[ d^{2} \right]=p_{C}v_{1}+\left( 1-p_{C} \right)v_{2}$; substitution gives the expression. (c) Solving (b) for $p_{C}$ with $\rho=c/p_{C}$. (d) The mean update is zero at $b=E\left[ s_{1}s_{2} \right]/E\left[ s_{1}^{2} \right]=p_{C}\rho$, and stochastic-approximation theory gives convergence. $▫$

*Remark (statistics of noisy measurements).* Proposition 2 concerns the values. If the observer registers $x_{i}=s_{i}+\varepsilon_{i}$ with independent noise of variance $\sigma_{i}^{2}$, the covariance of the measurements equals that of the values, $\mathrm{Cov}\left( x_{1},x_{2} \right)=p_{C}\rho\sigma_{p}^{2}$, while their variances are $\sigma_{p}^{2}+\sigma_{i}^{2}$, so that their correlation, $p_{C}\rho\sigma_{p}^{2}/\left[ \left( \sigma_{p}^{2}+\sigma_{1}^{2} \right)\left( \sigma_{p}^{2}+\sigma_{2}^{2} \right) \right]^{1/2}$, is attenuated by the reliabilities of the two channels. The measured difference $x_{2}-x_{1}$ is a scale mixture of Gaussians with variances $a=w^{2}+\sigma_{1}^{2}+\sigma_{2}^{2}$ under a common source and $b=2\sigma_{p}^{2}+\sigma_{1}^{2}+\sigma_{2}^{2}$ under independent sources, whose excess kurtosis is $\gamma_{2}=3p_{C}\left( 1-p_{C} \right)\left( b-a \right)^{2}/V^{2}$ with $V=p_{C}a+\left( 1-p_{C} \right)b$; it reduces to Proposition 2(b) without noise and vanishes exactly when $p_{C}\in\{0,1\}$ or $\rho=0$. With the noise variances known, $\sigma_{p}^{2}$ follows from the measured variances and $b$ from $\sigma_{p}^{2}$, and then $p_{C}=3\left( b-V \right)^{2}/\left[ 3\left( b-V \right)^{2}+\gamma_{2}V^{2} \right]$, which reduces to Proposition 2(c) without noise, and $\rho=Cov\left( x_{1},x_{2} \right)/\left( p_{C}\sigma_{p}^{2} \right)$. Noise therefore leaves both priors identified, but it dilutes the kurtosis, since it adds the same variance to both components, so that more observations are needed to estimate it with a given precision.

**Proposition 3 (common-cause prior and criterion).** Let a report of common source be issued with probability $g\left( L\left( \mathbf{x} \right)-logit\kappa\right)$ for any function $g$, which includes the deterministic rule $P\left( C=1\mid\mathbf{x} \right)>\kappa$ and its softened versions. Then the probability of every pattern of reports depends on $\left( p_{C},\kappa\right)$ only through $\mathrm{logit}p_{C}-logit\kappa$.

*Proof.* By Bayes' rule $L\left( \mathbf{x} \right)=logitp_{C}+logp\left( \mathbf{x}\mid C=1 \right)-logp\left( \mathbf{x}\mid C=2 \right)$, and the likelihoods do not depend on $p_{C}$. The argument of $g$ is therefore $\left( \mathrm{logit}p_{C}-logit\kappa\right)$ plus a function of $\mathbf{x}$ that does not involve either parameter. The result does not depend on the Gaussian assumptions. $▫$

**Proposition 4 (broad prior over values).** Holding $w$ fixed and letting $\sigma_{p}\to\infty$: (a) the weight of $x_{2}$ in the estimate of $s_{1}$ under a common cause tends to $\sigma_{1}^{2}/V_{1}$, and the weight of $x_{1}$ to $\left( w^{2}+\sigma_{2}^{2} \right)/V_{1}$, with $V_{1}=w^{2}+\sigma_{1}^{2}+\sigma_{2}^{2}$; (b) $\log\mathcal{N}\left( \mathbf{x};\mathbf{0},A_{1} \right)-log\mathcal{N}\left( \mathbf{x};\mathbf{0},A_{2} \right)=-\frac{\left( x_{2}-x_{1} \right)^{2}}{2V_{1}}+\frac{1}{2}\log\frac{\sigma_{p}^{2}}{V_{1}}+o\left( 1 \right)$; (c) with a deterministic criterion, the disparity at which a common source ceases to be reported satisfies $\Delta^{*2}=2V_{1}\left[ \mathrm{logit}p_{C}-logit\kappa+\frac{1}{2}\log\left( \sigma_{p}^{2}/V_{1} \right) \right]$, and $\partial\Delta^{*2}/\partial V_{1}>0$ if and only if $\mathrm{logit}p_{C}+\frac{1}{2}\log\left( \sigma_{p}^{2}/V_{1} \right)>logit\kappa+\frac{1}{2}$.

*Proof.* Substitute $\rho=1-w^{2}/\left( 2\sigma_{p}^{2} \right)$ in $\Sigma_{1}A_{1}^{-1}$ and in the two Gaussian log-densities and take limits; $\left| A_{1} \right|=\sigma_{p}^{2}V_{1}+O\left( 1 \right)$ and $\left| A_{2} \right|=\sigma_{p}^{4}+O\left( \sigma_{p}^{2} \right)$. Part (c) solves $L=logit\kappa$ in the limit form and differentiates. It follows from (a) and (b) that the estimate of $s_{1}$ and the posterior depend on $w^{2}$ and $\sigma_{2}^{2}$ only through $V_{1}$ and $\sigma_{1}^{2}$, and that $p_{C}$ and $\sigma_{p}$ enter the log-odds only through $\mathrm{logit}p_{C}+log\sigma_{p}$. $▫$

**Proposition 5 (fixed common source).** Let $p_{C}=1$, let the white, rosé and dyed white wines have (hue, odor) equal to $\left( -\delta,-\delta\right)$, $\left( \delta,\delta\right)$ and $\left( \delta,-\delta\right)$ in standardized units, and let $\hat{o}=a_{h}x_{h}+a_{o}x_{o}$ be the posterior mean of odor. Then $a_{h}=\rho\sigma_{p}^{2}\sigma_{o}^{2}/\left| A_{1} \right|$, $a_{o}=\sigma_{p}^{2}\left( \sigma_{p}^{2}\left( 1-\rho^{2} \right)+\sigma_{h}^{2} \right)/\left| A_{1} \right|$, and the displacement $D=\left( E\hat{o}_{\mathrm{dyed}}-E\hat{o}_{\mathrm{white}} \right)/\left( E\hat{o}_{rosé}-E\hat{o}_{\mathrm{white}} \right)=a_{h}/\left( a_{h}+a_{o} \right)$ equals $\rho\sigma_{o}^{2}/\left[ \rho\sigma_{o}^{2}+\sigma_{p}^{2}\left( 1-\rho^{2} \right)+\sigma_{h}^{2} \right]$. Moreover $\partial D/\partial\rho=\sigma_{o}^{2}\left( \sigma_{p}^{2}\left( 1+\rho^{2} \right)+\sigma_{h}^{2} \right)/\mathrm{den}^{2}>0$ and $\partial D/\partial\sigma_{o}^{2}=\rho\left( \sigma_{p}^{2}\left( 1-\rho^{2} \right)+\sigma_{h}^{2} \right)/\mathrm{den}^{2}>0$ for $\rho>0$, with $den=\rho\sigma_{o}^{2}+\sigma_{p}^{2}\left( 1-\rho^{2} \right)+\sigma_{h}^{2}$.

*Proof.* The weights are the second row of $\Sigma_{1}A_{1}^{-1}$. The expected estimates are linear in the stimulus values, so the differences reduce to $2\delta a_{h}$ and $2\delta\left( a_{h}+a_{o} \right)$. The derivatives follow by direct differentiation. The discriminability $d'=2\delta\left( a_{h}+a_{o} \right)/\sqrt{a_{h}^{2}\sigma_{h}^{2}+a_{o}^{2}\sigma_{o}^{2}+\sigma_{m}^{2}}$ of rosé and white, with report noise $\sigma_{m}$, increased with $\rho$ at every point of a grid spanning $\rho\in\left[ 0.05,0.95 \right]$, $\sigma_{o}\in\left[ 0.3,2 \right]$, $\sigma_{h}\in\left[ 0.02,0.2 \right]$ and $\sigma_{m}\in\left[ 0,0.6 \right]$ ($\sigma_{p}=1$). The increase can fail near $\rho=1$ when hue noise is comparable with or larger than odor noise, a regime outside this grid and outside the case of clearly distinct hues. $▫$

*Remark (the variances in expression 6).* With hue and odor expressed on a common standardized scale, both have variance $\sigma_{p}^{2}$ across wines and correlation $\rho$, so that $E\left[ o\mid h \right]=\rho h$ and the conditional variance of odor given hue is $\mathrm{Var}\left( o\mid h \right)=Var\left( o \right)-Cov\left( o,h \right)^{2}/Var\left( h \right)=\sigma_{p}^{2}-\rho^{2}\sigma_{p}^{4}/\sigma_{p}^{2}=\sigma_{p}^{2}\left( 1-\rho^{2} \right)$, the variation of odor among wines of the same hue. The ratio of the two weights is $a_{o}/a_{h}=\left[ \sigma_{p}^{2}\left( 1-\rho^{2} \right)+\sigma_{h}^{2} \right]/\left( \rho\sigma_{o}^{2} \right)$: the hue channel is down-weighted in proportion to $\sigma_{p}^{2}\left( 1-\rho^{2} \right)+\sigma_{h}^{2}$, the conditional variance plus the visual noise, which is proportional to the uncertainty about odor that remains once hue has been registered, $Var\left( o\mid x_{h} \right)=\sigma_{p}^{2}\left[ \sigma_{p}^{2}\left( 1-\rho^{2} \right)+\sigma_{h}^{2} \right]/\left( \sigma_{p}^{2}+\sigma_{h}^{2} \right)$. The noise of hue therefore matters only when $\sigma_{h}^{2}$ is comparable with $\sigma_{p}^{2}\left( 1-\rho^{2} \right)$, which for clearly distinct hues, whose visual noise is small, requires a mapping from hue to odor close to deterministic.

# Supplementary Material S2: Simulation and fitting methods

*Observer and data.* Simulated observers followed the model stated at the beginning of Supplementary Material S1. The probability of a report of common source was $\lambda/2+\left( 1-\lambda\right) \mathrm{logistic}\left( \beta\left( L\left( \mathbf{x} \right)-logit\kappa\right) \right)$, with decision slope $\beta=3$ and lapse rate $\lambda=0.02$ fixed; continuous estimates of $s_{1}$ carried Gaussian report noise $\sigma_{m}$. Unimodal reports of channel $i$ were Gaussian with mean $k_{i}s_{i}$ and variance $k_{i}^{2}\sigma_{i}^{2}+\sigma_{m}^{2}$, where $k_{i}=\sigma_{p}^{2}/\left( \sigma_{p}^{2}+\sigma_{i}^{2} \right)$. The bimodal battery crossed five positions of $s_{1}$ (−6, −3, 0, 3, 6 units) with nine disparities (0, ±2, ±5, ±10, ±18) and was repeated 5, 10, 20 or 40 times, giving 225 to 1800 bimodal trials, with 10 unimodal estimation trials per position and channel and two-interval discrimination trials for each channel (comparison offsets of ±1, ±2, ±4 and ±8 units for channel 1 and ±0.5, ±1, ±2 and ±4 units for channel 2, 15 trials each), whose probability of judging the comparison larger was $\lambda/2+\left( 1-\lambda\right)\Phi\left( \Delta/\left( \sqrt{2}\sigma_{i} \right) \right)$. The baseline observer had $p_{C}=0.5$, $w=2$, $\sigma_{1}=3$, $\sigma_{2}=1$, $\sigma_{p}=10$, $\sigma_{m}=1$ and $\kappa=0.5$ (illustrative values in arbitrary units).

*Likelihood and fitting.* The likelihood of each bimodal trial, which jointly contains the estimate and the report generated by the same measurements, was computed by two-dimensional Gauss-Hermite quadrature over measurement noise with $96\times96$ nodes; refits of six diagnostic datasets, spanning the baseline, both trained states and extreme combinations of the two priors, agreed with a $128\times128$ reference within tolerances of 0.01 on the negative log-likelihood and on $p_{C}$ and $w$ and of 0.005 on $\kappa$. A floor of 0.5 on the report noise, below its generating range of 0.6 to 1.5, prevents spuriously narrow report kernels from exploiting the quadrature grid. Parameters were estimated on unconstrained scales (logit for $p_{C}$ and $\kappa$, logarithm for $w$ and the standard deviations) by L-BFGS-B with bounds and gradients from automatic differentiation (JAX), from three fixed starting points that did not depend on the generating values, keeping the best solution, with the coupling width kept within its valid range ($w<2\sigma_{p}$). For model recovery, the nested models were also evaluated at their exact limits ($w=0$ or $p_{C}=1$) and embedded in their parent models, and the solution with the smallest negative log-likelihood was retained, so that a finite fitting bound is never read as an exact special case. Some fits were initialized from solutions obtained with fewer quadrature nodes and then optimized with the 96-node objective; the replication package documents these starts and the direct multistart comparisons that check them.

*Recovery.* For parameter recovery, generating values were drawn uniformly from $p_{C}\in\left[ 0.2,0.8 \right]$, $\log w\in\left[ \log0.5,log4 \right]$, $\sigma_{1}\in\left[ 2,4 \right]$, $\sigma_{2}\in\left[ 0.6,1.5 \right]$, $\sigma_{p}\in\left[ 7,14 \right]$, $\sigma_{m}\in\left[ 0.6,1.5 \right]$ and $\kappa\in\left[ 0.3,0.7 \right]$, with 30 datasets per trial count. For model recovery, 20 datasets of 900 bimodal trials were generated from each of forced fusion ($p_{C}=1$, $w=0$), the coupling-prior model ($p_{C}=1$, $w=2$), causal inference ($p_{C}=0.5$, $w=0$) and the full model ($p_{C}=0.5$, $w=2$); all four models were fitted to the continuous estimates and the unimodal data, and the model with the lowest BIC was selected, with the criterion $\kappa$, which does not affect continuous estimates, excluded from the parameter count. The two-prior model and causal inference, which both produce common-source reports, were also compared on the full dual-report battery, with 20 datasets generated from each of the two-prior model with $w=2$ and $w=3$ and from causal inference. With the continuous estimates and the unimodal data alone, forced fusion, the coupling-prior model and causal inference were each selected in all 20 of the datasets that they generated (Wilson 95% interval 84 to 100%), and data from the two-prior model with $w=2$ were attributed to causal inference in 12 of 20 (60%; 39 to 78%). Reduced designs removed the continuous estimates, the unimodal trials of channel 2, or the disparities beyond ±5 units (with the number of bimodal trials held at 900), with 25 datasets each.

*Power.* For the two-protocol design, in which one training protocol lowers $p_{C}$ and the other narrows the coupling width $w$, participants were tested before and after each protocol with the full battery of 900 bimodal trials per session. Protocol A lowered $p_{C}$ from 0.5 to 0.3 and reduced the sensory noise of both channels by 15%; protocol B halved the coupling width. The analysis was repeated with a more precise target channel ($\sigma_{1}=2$ before training). True changes varied across participants with a standard deviation equal to half of the predicted change, and estimation errors were resampled from 30 fits at each state. The predicted dissociation was tested with paired contrasts across protocols on each parameter, on the logit scale for $p_{C}$ and on the natural scale for $w$, whose null value lies on the boundary of its range (two-sided $\alpha=0.05$, with the effect required in the predicted direction), over 4,000 simulated experiments per sample size. A noncentral-$t$ approximation that includes the mean and variance of the estimation errors reproduced the simulated power.

*Reduced designs.* With common-source reports alone, the likelihood was exactly flat along the line on which logit $p_{C}$ − logit $\kappa$ is constant, whereas with the continuous estimates added, shifts of −1 and +1 logit units along that line raised the negative log-likelihood by 17.6 and 20.8 units (Supplementary Figure S1A). Without common-source reports, $p_{C}$ remained identified by the estimates, with a standard deviation of logit $p_{C}$ across simulated observers of 0.35 against 0.27 with the full battery. Without unimodal trials for the influencing channel, the coupling width and the noise of that channel traded against each other (correlation of the fitted values −0.49), the standard deviation of the coupling width rose to 0.89 units, and $\sigma_{2}$ was underestimated. When the precision of each channel was measured only through the variability of unimodal estimates (no discrimination trials, otherwise identical design), $\sigma_{2}$ was recovered with a correlation of 0.69 and reached the lower bound of its range in 6 of 30 datasets, and the coupling width was overestimated by 0.15 units on average, against a mean error of −0.03 units with the discrimination trials, because unimodal estimation variance mixes sensory noise with report noise. With disparities limited to ±5 units and the same number of trials, both priors were estimated with similar precision, but the correlation between their estimates rose from 0.34 to 0.61.

*Model recovery with the full battery.* The two-prior model and causal inference were compared on the full dual-report battery with 20 datasets per generating model. The two-prior model was preferred in 70% of datasets generated with $w=2$ (median BIC advantage 2.5) and in 100% of those generated with $w=3$ (median advantage 17.7), and causal inference was retained in 100% of the datasets that it generated (Wilson 95% intervals 48 to 86%, 84 to 100% and 84 to 100%).

*Prior width and the common-cause prior.* Since $p_{C}$ and $\sigma_{p}$ share the intercept of the log posterior odds (Proposition 4), errors in their estimates may compensate. The correlation between the estimation errors of $\log\sigma_{p}$ and $\mathrm{logit}p_{C}$ was -0.19 at the baseline state, -0.24 and -0.07 after the two protocols, and -0.02 across the range of generating values, while the mean error of $\mathrm{logit}p_{C}$ stayed between -0.03 and -0.00, so that the misestimation of $\sigma_{p}$ adds variance to $p_{C}$ without biasing it.

*A more precise target channel.* With $\sigma_{1}=2$ before training, 7 of 30 fits still placed the coupling at the lower bound of the fitting range after protocol B, and 80% power for the $p_{C}$ contrast required 15 participants (against 10 with $\sigma_{1}=3$), since a precise target channel reduces the influence of the other signal on its estimate (amplitude 0.26 against 0.33) and with it the information that the estimates carry about the posterior over causal structures.

*Robustness to the read-out.* The alternative read-outs were checked against the model-averaging observer (the per-structure estimates reproduce it exactly, all read-outs coincide when the posterior is 0 or 1, and probability matching is unbiased for the averaging estimate). Simulated observers were generated with model selection (the estimate of the more probable structure) or probability matching (a structure drawn with its posterior probability) and fitted with the model-averaging observer, with 20 datasets per state. With probability matching, the fitted changes were -0.74 in logit $p_{C}$ after protocol A (true -0.85) and -0.94 units in $w$ after protocol B (true -1), with cross-changes of 0.27 and 0.33, and 80% power for the double dissociation required 15 participants. A replication with fresh random seeds gave -0.94 in logit $p_{C}$ after protocol A and -0.87 units in $w$ after protocol B, cross-changes of 0.19 and 0.51, and 80% power for the double dissociation with 10 participants, so that the sample needed lies between 10 and 15 at the resolution of the simulated grid; the spurious widening of $w$ after protocol A amounts to a third to a half of the true narrowing after protocol B. Under probability matching the fitted coupling was also biased in absolute terms: its mean before training was 0.96 and 0.89 in the two runs for a true value of 2, and every fit after protocol B reached the lower bound for a true value of 1. With model selection, the fitted change in $p_{C}$ after protocol A was exaggerated (-1.91), the fitted coupling widened after protocol A (1.51 units) and did not narrow after protocol B (0.00), so that the contrast on $w$ reached significance for the wrong reason; the replication gave -1.94, 1.12 and 0.00 for the same three quantities. The read-out of each observer must therefore be established by comparing fits with each read-out before parameters are compared across sessions.

*Assumptions of the power analysis.* The simulated experiments assume that neither protocol carries over into the other and that the estimation errors observed at the mean parameter values apply to every participant; protocol order must be counterbalanced with a washout between protocols.

*Code.* The code, the configuration and the random seeds that reproduce every number and figure are provided with the submission as a replication package and will be deposited in a public repository with a persistent identifier upon acceptance. Enquiries about the software can be addressed to Fabio Lokwani-Di Matteo (fabio.lokwani-dimatteo@phd.unich.it).

# Supplementary Material S3: Sensitivity of the power estimates

The power curves of Figure 4E draw on 30 fitted estimation-error records at each state, and their Monte Carlo intervals leave aside the uncertainty of that finite library. Two further analyses examine it without new fits. First, 300 outer libraries were formed by resampling the 30 complete fit records with replacement within each state, which keeps the joint errors and biases of each record, and 4,000 experiments were simulated for each library, sample size (10, 15, 20, 30 and 40 participants) and target-noise regime. Second, the Gaussian spread of the true coupling widths, which assigns a probability of 0.023 to a negative width after protocol B, was replaced by scaled Beta distributions on the valid range $0\leq w\leq2\sigma_{p}$ with the same mean and variance and, in a third scenario, the saved estimation errors were also transported in coordinates that respect that range. The contrasts and the significance rule were those of section 7.

With the nominal library, power for both contrasts together at 15 participants was 0.938 to 0.948 across the three scenarios for the baseline target and 0.833 to 0.836 for the more precise target, so that 15 remained the smallest tested sample reaching 0.80 in every scenario and regime. Resampling the library produced much more variation than the change of scenario: with coordinate transport and 15 participants, the 2.5th to 97.5th percentiles of conjunction power across libraries were 0.819 to 0.974 for the baseline target and 0.560 to 0.939 for the more precise target, and 295 to 297 of the 300 baseline libraries, against 176 to 182 of the 300 precise-target libraries, reached 0.80; with 20 participants the bands were 0.928 to 0.997 and 0.727 to 0.988 (Supplementary Figure S4). The predicted dissociation is therefore robust to the form of the distribution of coupling changes, whereas the sample it requires depends on the finite library of fitting errors, especially when the target channel is precise. These bands are conditional design benchmarks, not confidence intervals for the power of a future study.

# Supplementary Material S4: Participant-level reanalysis of the wine descriptor data

The data of Wang and Spence (2019) are public (Q. J. Wang, 2019, Mendeley Data, V1, doi: 10.17632/jxx5d7n96r.1, licensed under CC BY 4.0), which allows a direct check of the aroma pattern that motivates the chemosensory example. The analysis uses the aroma descriptors that each participant chose for the white wine (W), the rosé (R) and the white dyed to resemble the rosé ($R'$), for the 79 self-reported experts and the 22 beginners; the deposit contains 169 records against the 168 reported in the article, the difference lying in the intermediate group, which this contrast does not use. Counts of the 30 individual descriptors reproduce all 12 aroma coefficients and 23 of the 24 coefficients of the article's Table 7 at their reported precision, whereas the seven descriptor categories reproduce one, so the 30 descriptors provide the primary representation.

For each group, let $F_{W}$, $F_{R}$ and $F_{R'}$ be the summed descriptor counts and $s\left( \cdot,\cdot\right)$ the cosine similarity between two profiles. The contrast $T=\left[ s\left( F_{R},F_{R'} \right)-s\left( F_{W},F_{R'} \right) \right]_{\mathrm{experts}}-\left[ s\left( F_{R},F_{R'} \right)-s\left( F_{W},F_{R'} \right) \right]_{\mathrm{beginners}}$ is positive when the profile of the dyed white lies closer to the rosé, relative to the white, for experts than for beginners. It concerns group descriptor profiles, not individual judgments or fitted parameters of the model. Intervals come from 20,000 bootstrap samples drawn within each group, with each sampled participant carrying the responses to all three wines (Efron, 1979). Two sensitivity analyses were fixed before the intervals were computed: the seven published descriptor categories, and the participants who recorded all nine aroma descriptors (67 experts and 18 beginners).

The primary contrast was $T=0.321$, with a 95% interval from $-0.035$ to $0.639$; with the seven categories it was 0.399 (0.100 to 0.720), and among complete reporters 0.291 ($-0.083$ to 0.657) (Supplementary Figure S5). The published direction is reproduced, with an uncertainty that includes zero in two of the three specifications. The limits of the data bear on this reading: expertise was self-reported, the groups were unequal and recruited at one event, the dyed wine was always presented last, and unreported descriptors may differ from reported ones. The analysis estimates the descriptive evidence that motivates the model; it does not fit the parameters of the model to human observers.

# Supplementary Figures

**Supplementary Figure S1.** Contributions of each component of the battery. (A) Negative log-likelihood of one simulated dataset along the line on which logit $p_{C}$ − logit $\kappa$ is constant, with the other parameters at their maximum-likelihood values: exactly flat with common-source reports alone (blue), as Proposition 3 requires, and curved when continuous estimates are added (black). (B) Fitted coupling width and noise of the influencing channel with (black) and without (orange) unimodal trials for that channel. (C) Recovery of $\sigma_{2}$ when precision is measured by the variability of unimodal estimates only (grey), which mixes sensory with report noise, and with discrimination trials added (purple). (D) Fitted $p_{C}$ and $w$ with disparities up to 18 units (black) and up to 5 units only (green). (E) Fitted logit $p_{C}$ for a baseline observer and for the same observer after a pure increase in precision, with and without unimodal trials. (F) Relative error of the recovered coupling width as a function of its ratio to the combined sensory noise.

**Supplementary Figure S2.** Lessons from an environment. A covariance (delta-rule) learner (A) and a mixture learner (B, C) exposed to an environment with rare common sources and tight coupling ($p_{C}=0.3$, $\rho=0.95$) and to an environment with a common source on every event and loose coupling ($p_{C}=1$, $\rho=0.285$), matched on the product $p_{C}\rho$ (dashed line). Medians and interquartile ranges over 40 simulated learners.

**Supplementary Figure S3.** Parameter recovery for all seven free parameters at 900 bimodal trials.

**Supplementary Figure S4.** Sensitivity of the power estimates. Power for the contrast in $p_{C}$ (A, D), the contrast in $w$ (B, E) and both together (C, F) as a function of the number of participants, for the baseline target channel (top) and a more precise target channel (bottom). Lines show the nominal library of fitting errors with Gaussian coupling widths and additive error transport, Beta widths with additive transport, and Beta widths with transport in valid coordinates (the $p_{C}$ contrast is common to the three scenarios); shaded bands show the 2.5th to 97.5th percentiles across 300 resampled error libraries. The dotted line marks a power of 0.80.

**Supplementary Figure S5.** Participant-level reanalysis of the wine descriptor data. Expert-minus-beginner contrast $T$ in how much closer the aroma profile of the dyed white lies to the rosé than to the white, with 95% bootstrap intervals, for the primary analysis (30 descriptors, all recorded descriptors), the seven published descriptor categories, and the participants who recorded all nine aroma descriptors.
